# Reprogramming myeloid cells and restoring T cell fitness in checkpoint inhibitor resistant melanoma patients

**DOI:** 10.1186/s40364-026-00917-z

**Published:** 2026-03-27

**Authors:** Hanna Grauers Wiktorin, Viktoria Ekström-Rydén, Ida Ek, Emma Eriksson, Tanja Lövgren, Clara Bernedal Nordström, Linda C. Sandin, Meera R. Patel, Omid Hamid, Gustav Ullenhag, Angelica Loskog

**Affiliations:** 1 Department of Immunology, Genetics and Pathology (IGP), Uppsala University, Uppsala, Sweden; 2 Department of Oncology, Uppsala University Hospital, Uppsala, Sweden; 3 Lokon Pharma AB, Uppsala, Sweden; 4 Baylor College of Medicine, McNair Campus, Houston, TX USA; 5 The Angeles Clinic and Research Institute, A Cedars Sinai Affiliate, Medical Oncology, Los Angeles, CA USA

**Keywords:** Malignant melanoma, Gene engineering, Immune checkpoint inhibitors, Dendritic cells, CD8^+^ T cells, Immunotherapy, Tumor microenvironment, Immune checkpoint inhibitor resistance, CD40L, 4-1BBL

## Abstract

**Background:**

Immune checkpoint inhibitor (ICI) resistance remains a clinical hurdle in malignant melanoma (MM). Remodeling the tumor microenvironment (TME) have been proposed a revenue to overcome ICI resistance. The present study aimed to test the hypothesis that treatment with LOAd703, a TMZ-CD40L/4-1BBL-expressing gene engineering vector, reprograms the immune landscape in ICI refractory MM patients to restore ICI responsiveness.

**Methods:**

LOKON003 a multi-center, single-armed phase I/II clinical trial recruited 24 patients with confirmed stage IV cutaneous MM (*n* = 23) and mucosal MM (*n* = 1) who had progressed on one, or several, treatments with anti-PD-1 inhibitors with or without supplementary anti-CTLA-4 inhibitor or experimental immunotherapy treatment. LOAd703 (1 × 10^11^ or 5 × 10^11^ viral particles) was administered by ultrasound guided intratumoral injections and atezolizumab was administered intravenously (1200 mg). Patients were treated every third week for a maximum of 12 LOAd703/anti-PD-L1 treatments cycles, possibly followed by up to seven single treatments with atezolizumab. Peripheral blood mononuclear cells (PBMCs), plasma, and tumor biopsies were collected before and at nine (PBMC, plasma, and tumor biopsies), 18 (plasma), and 27 (tumor biopsies) weeks post treatment onset. Tumor biopsies were examined for expression of 770 immune and oncology related genes by NanoString, PBMC samples were immunophenotyped by flow cytometry, and proteomic analysis of 172 immune and oncology markers were assessed by Olink.

**Results:**

The results suggest that treatment with LOAd703 and atezolizumab renders a local and systemic immune signature in ICI resistant MM patients resembling what has previously been reported correlative with ICI response. This includes an increased TME expression of DC-associated biomarkers, an enhanced level of circulating CD40^+^ DC-like cells, an increase in biomarkers associated with T cell infiltration and T cells in the TME, elevation of circulating CD8^+^ T cells with an EM phenotype, and reduced circulating levels of CD4^+^ T cells including Tregs.

**Conclusions:**

Collectively, the results propose that treatment with LOAd703 and atezolizumab induce an immune phenotype of ICI resistant patients that has previously been reported associative with ICI responsiveness. These results merits further clinical investigation of supplementary LOAd703 treatment to accomplish ICI sensitivity in ICI resistant MM.

**Trial registration:**

Clinicaltrials.gov ID, NCT04123470. First posted 2019-10-11.

**Supplementary Information:**

The online version contains supplementary material available at 10.1186/s40364-026-00917-z.

## Background

The introduction of immune checkpoint inhibitors (ICIs) has revolutionized the treatment of metastatic cutaneous malignant melanoma (CMM). Despite this significant breakthrough, patients with metastatic CMM carry a 5-year survival rate of only 35% [1]. Combined therapy with anti-PD-1 and anti-CTLA-4 antibodies reportedly carries a 5-year survival rate to 49% [2] but at the cost of increased occurrence of immune-related adverse events and treatment-related toxicity [3]. Primary and secondary resistance to ICIs is common [2] and the main reason for treatment failure. This warrants investigation of means to restore immune capacity in resistant MM patients to achieve or reintroduce ICI sensitivity. Immune checkpoint inhibitory resistance may be tumor intrinsic and related to changes in processing and presentation of neoantigens [4, 5] or to oncogenic signaling resulting in reduced recruitment of lymphocytes to the tumor microenvironment (TME) [6]. Factors attributed to exploitation of immune checkpoints [5, 7] or establishment of an immunosuppressive TME [8] may also promote ICI resistance.

Tumor microenvironment gene engineering, defined by intratumoral delivery of an immunomodulatory gene cargo, represents one approach to reprogram the TME to support anti-tumor immunity which has been demonstrated to overcome murine ICI resistance [9]. LOAd703 (delolimogene mupadenorepvec) is an adenovirus-based genetic vector that introduces Th1 type immunostimulatory transgenes into the TME [10]. Viral replication has been tailored to depend on a dysregulated retinoblastoma pathway, a common event in malignant cells. Consequently, viral replication selectively occurs in tumor cells with subsequent oncolysis accompanied by the release of viral particles, danger signals, and tumor-associated and/or neoantigens. Transgene expression is, however, independent of viral replication and successively occurs in all infected cells, healthy as well as malignant [9, 10]. The vector design aims to introduce transgene expression in a larger proportion of the injected tumor lesion with prolonged duration compared to similar replication competent viral vectors.

LOAd703 encodes trimerized, membrane-bound CD40 ligand (TMZ-CD40L) and 4-1BB ligand (4-1BBL) [10]. CD40L is a pivotal mediator of anti-tumor immunity through its involvement in maturation and activation of dendritic cells (DCs), induction of receptors on endothelial cells that facilitate T cell attachment and transmigration into the TME, and production of immunoregulatory cytokines and chemokines [10, 11]. Combined stimulation of DCs by TMZ-CD40L, danger signals, and TLR9 engagement from the adenovirus are proposed to trigger superior DC maturation characterized by increased MHC I/II and costimulatory expression, improved capacity to produce IL-12 (favoring Th1 polarization), upregulation of CCR7 (supporting DC migration to lymph nodes to drive systemic immune responses), and improved DC presentation of neoantigens [12]. Subsequently resulting in improved anti-tumor T cell activation. Stimulation of activated T cells via 4-1BB is known to enhance the proliferation, survival, and effector functions of CD8^+^ T and NK cells [13]. LOAd703 is thus postulated to provoke anti-tumor immunity by boosting numerous aspects of innate and adoptive immunity. Preclinical work also indicates increased expression of PD-L1 on LOAd703 activated DC and synergistic effects between LOAd703 and PD-L1 blockade in preclinical cancer models have been reported [9, 12].

In this study, an extensive, hypothesis-driven post-hoc biomarker analysis was performed to define the mechanistic action of LOAd703 in combination with PD-L1 blockade in ICI refractory MM patients. Genetic, proteomic, and cellular analysis of pre- and post-treatment tumor biopsies and blood samples were evaluated for effects on immunomodulatory genes, proteins, and immune cells.

## Methods

### Study and sample description

LOKON003 (NCT04123470) was a multi-center, single-armed phase I/II clinical trial conducted between 2021 and 2023. The study was designed to evaluate the safety of combined LOAd703 and atezolizumab treatment. LOKON003 intended to enroll at least twenty-five and a maximum of thirty-five patients. Twenty-six patients were screened for eligibility, of which twenty-four were enrolled. Twenty-three patients had confirmed stage IV cutaneous MM (*n* = 23) and one mucosal MM (*n* = 1). All had progressed on one, or several, treatments with anti-PD-1 inhibitors with or without supplementary anti-CTLA-4 inhibitor or experimental immunotherapy treatment. Patients were followed for at least a year post the last LOAd703 and atezolizumab administration or until death or withdrawn consent, whatever came first.

In brief, LOAd703 was administered by ultrasound guided intratumoral injections. Seven patients received 1 × 10^11^ and seventeen 5 × 10^11^ viral particles. Atezolizumab was administered intravenously (1200 mg). Patients were treated every third week for a maximum of 12 LOAd703/anti-PD-L1 treatments cycles, followed by up to seven single treatments with atezolizumab. No power calculation was conducted to calculate the acquired sample size to define the immunological effects of LOAd703 and atezolizumab therapy as the power calculation was based on expected effect. This study is based on post-hoc tests.

Peripheral blood mononuclear cells (PBMCs), plasma, and tumor biopsies were collected before and at nine (PBMC, plasma, and tumor biopsies), 18 (plasma), and 27 (tumor biopsies) weeks post treatment onset. PBMCs were analyzed by flow cytometry, plasma by Olink proteomics, and bulk mRNA from tumor biopsies by NanoString transcriptomics. PBMC samples from five patients were not analyzed by flow cytometry due to bad quality of cells. In addition, one patient was excluded from the proteomics analysis based on poor protein detection throughout all analytes in one of the two proteomic panels. Figures show each individual data point when possible.

### LOAd703 and atezolizumab

LOAd703 was produced under Good Manufacturing Practice at Baylor College of Medicine’s Center for Cell and Gene Therapy, Houston, TX. Atezolizumab was provided by Hoffmann-La Roche.

### Ethics

The ethical boards of all treating affiliations approved the study which was conducted in accordance with the declaration of Helsinki and ICH Harmonized Tripartite Guideline for Good Clinical Practice. All patients gave written informed consent before inclusion and had the right to withdraw consent at any time.

### Flow cytometry

Cryopreserved PBMCs collected before (*n* = 19) and at nine (*n* = 14) weeks post-treatment onset were thawed and stained with Live Dead Zombie Near IR (Biolegend) following staining with five (referred to as Panle A-E) fluorescently labeled antibody panels (Supplementary Table 1).

Cells were acquired on a three-laser (405 nm, 488 nm, and 633 nm) BD Canto II (BD Biosciences, RRID: SCR_018056). Data was analyzed using FlowJo version 10.10.0 or later (RRID: SCR_008520) and FlowAI employed for quality control. Data was processed either by traditional hierarchical gating strategies (Panel D-E) or by unsupervised clustering using Phenograph which was visualized by tSNE (Panel A-C). For Panel A, live cells from each sample were downsized to 10 000 events, samples were concatenated and Phenograph employed for cluster analysis. Phenograph identified 29 clusters which were manually pooled into 11 population visualized by tSNE. For Panel B and C, 5000 live CD3^+^ cells were downsampled from each sample. One concatenated file was created for each panel. For Panel B traditional gating was used to identify naïve, effector memory, central memory, and Temra of CD4^+^ and CD8^+^ T cells. For panel C, phenograph identified 31 clusters that were manually pooled into 5 populations; conventional CD4^+^ T cells, regulatory T cells, CD8^+^ T cells, CD4^+^CD8^+^ (double positive: DP), and CD4^−^CD8^−^ (double negative: DN) T cells visualized by tSNE.

### Olink

The Olink Target 96 Immuno-Oncology panel and the Olink Target 96 Oncology II panel (OlinkProteomics AB, Uppsala, Sweden, RRID: SCR_003899) were used for proteomic analysis of plasma harvested at onset (*n* = 24), at nine (*n* = 17), and 18 (*n* = 13) weeks post treatment initiation. Samples were analyzed at TATAA Biocenter, Gothenburg, Sweden. Data is presented as log_2_ normalized protein expression (NPX).

### NanoString

Tumor biopsies were collected at baseline (*n* = 16) and at week nine (*n* = 14), 12 (*n* = 1), and 27 (*n* = 10) post treatment initiation. From tumor biopsies, mRNA was purified using RNeasy Mini Kit (QIAGEN, Hilden, Germany) according to manufacturer’s instructions. The mRNA from each patient was analyzed with the NanoString technology (NanoString Technologies, Seattle, WA, USA, RRID: SCR_023912) using the nCounter^®^ PanCancer Immune Profiling Panel at the Clinical Genomics Unit at the Uppsala University, Sweden. Data was normalized with RUV-4 in R Studio (version 2024.12.0 + 467) and R (version 4.2.1, RRID: SCR_001905) for the fold change between baseline and week nine or week 27. The approximant individual value was calculated by removing estimated unwanted variation calculated during the RUV-4 normalization from the observed log_2_ gene count.

### Statistics

Wilcoxon paired singed-rank test was used to compare differences between pre- and post-treatment levels of immune cells in PBMC samples. Heatmaps are presented as fold change (post-treatment levels/pre-treatment levels). Harmonic mean p-values (HMP) were calculated accordingly: (sum of reciprocal p-values)/n. Differences in plasma protein levels for each protein panel were determined using the Benjamini Hochberg test with a false discovery rate (FDR) of 5%. Transcriptomic analysis in the TME was based on gene expression of 156 immune cell-related genes extracted from the 770 genes in the nCounter^®^ PanCancer Immune Profiling Panel. Nominal p-values for treatment induction of the 156 genes were adjusted for multiple comparison using the Benjamini Hochberg test with a FDR of 5%. K-means clustering was performed using Jamovi version 2.3.28 or later (RRID: SCR:016142). Univariate and multivariate Cox regression analysis was conducted in R Studio (version 2024.12.0 + 467) and R (version 4.2.1, RRID: SCR_001905). Parameters that reached a p-value below 0.05 for gene expression in the TME or 0.1 for circulating levels of immune cells in univariable Cox regression analysis were included in multivariable Cox regression analysis. Survival analysis was performed by the Log rank Mantel-Cox test to compare the impact of immune cell populations on overall survival (OS) (patients were dichotomized by median).

## Results

### Increased expression of genes associated with dendritic cells and antigen presentation in the TME following LOAd703 and atezolizumab treatment

**Fig. 1:**
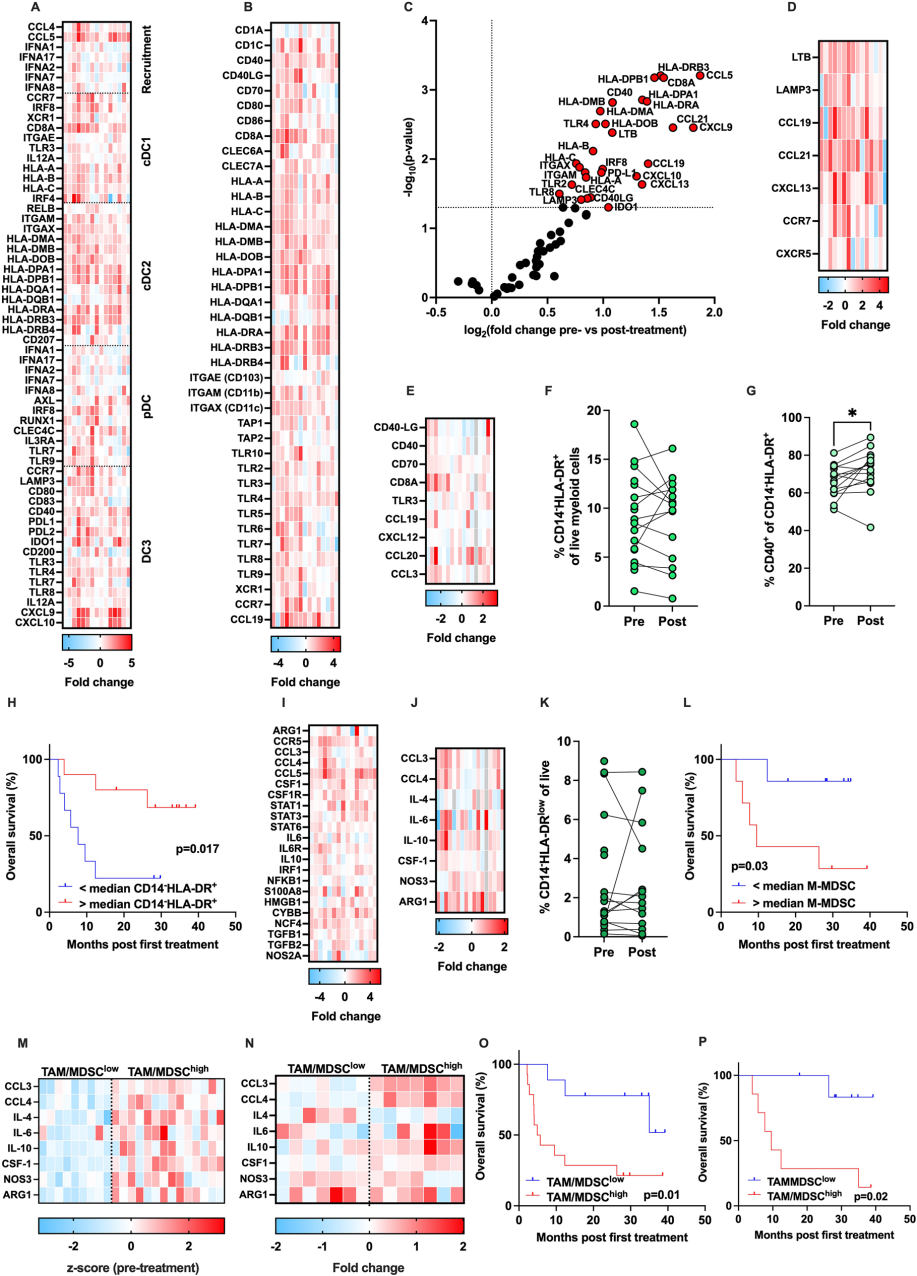
Combined therapy with LOAd703 and atezolizumab induces increased expression of genes associated with dendritic cells and antigen presentation in the tumor microenvironment. Gene expression in tumor biopsies at baseline (pre) and at nine weeks post treatment induction (post) were measured using NanoString. (**A**-**B**) Fold change, post versus pre, of gene expression associated with dendritic cells (DCs; **A**) and antigen presentation (**B**). (**C**) Volcano plot of genes associated with DCs, antigen presentation, and tertiary lymphoid structures (TLS) in the tumor microenvironment in pre- and post-treatment (week nine) tumor biopsies. Red indicates genes more highly expressed post-treatment compared with pre-treatment. Statistics by paired *t* tests with correction for multiple comparison by the false discovery rate (5%) method of Benjamini Hochberg. (**D**) Fold change, nine weeks post-treatment initiation versus baseline, of genes associated with TLS. Protein expression in plasma was evaluated with the Olink 96 target Immuno-oncology and Oncology II proteomics panels before (pre) and nine weeks after (post) treatment onset. (**E**) Fold change, post versus pre, protein expression of DC-associated proteins. (**F**-**G**) Alterations of circulating myeloid cells were assessed at baseline (pre) and nine weeks (post) after treatment initiation by flow cytometry. Pre- and post-treatment frequencies of CD14^-^HLA-DR^+^ (**F**) and CD14^-^HLA-DR^+^CD40^+^ (**G**) cells among peripheral blood mononuclear cells. Statistics by Wilcoxon. (**H**) Kaplan-Meier curve of patients based on an above or below median frequency of CD14^-^HLA-DR^+^ cells of live cells at treatment onset. Statistics by the log-rank Mantel Cox test. (**I**-**J**) Fold change, post versus pre, of genes (**I**) and proteins (**J**) associated with tumor associated macrophages (TAMs) and myeloid-derived suppressor cells (MDSCs). (**K**) Pre- and post-treatment frequencies of myeloid MDSCs defined as CD14^+^HLA-DR^low^ cells out of live cells. (**L**) Kaplan-Meier curve of patients based on an above or below median frequency of CD14^+^HLA-DR^low^ cells of live cells at nine weeks after treatment initiation. Statistics by the log-rank Mantel Cox test. (**M**-**N**) Heatmaps of K-mean clusters of MDSC-associated protein levels at baseline, displayed as z-score (**M**) and fold change (**N**). Kaplan-Meier curve of patients in the MDSC^low^ and MDSC^high^ clusters at baseline (**O**) and of fold change (**P**). Statistics were conducted by the Log rank Mantel-Cox test. Results from gene expression and protein levels are generated from 15 and 17 paired pre- and post-treatment samples, respectively. * *p* < 0.05

Patients were treated every third week with LOAd703 intratumoral injections and intravenous atezolizumab. mRNA was purified from tumor biopsies collected from the injected tumor lesion at baseline and at nine and 27 weeks post treatment initiation and analyzed by the NanoString technology. A profound induction of genes associated with DC and antigen presentation signatures in the TME was observed after treatment initiation (Fig. 1A-C, Supplementary Table 2, Supplementary Fig. 1A-C, and Supplementary Table 3). In an attempt to test if the entire set of DC- and antigen presentation-associated genes were altered by the treatment, the harmonic mean p-value (HMP) was calculated. Both the DC (HPM = 0.00025 and HPM = 0.0015) and the antigen presentation (HMP = 0.00031 and HMP = 0.0025) gene signatures were significantly induced at week nine and 27, respectively.

Several tertiary lymphoid structure (TLS)-associated genes were also upregulated in the TME at week nine (Fig. 1C-D) and 27 (Supplementary Fig. 1C-D) with HMP of 0.0023 and 0.0024, respectively, indicative of treatment-enhanced aggregation of immune cells and on-site anti-tumor immune activation. At week 27, CCR7 was induced (*p* = 0.0054, *n* = 10, paired *t* tests with correction for multiple comparison by the FDR (5%) method of Benjamini Hochberg), possibly supporting ongoing migration towards draining lymph nodes. To further elaborate on a potential systemic DC activation, plasma samples collected at baseline and at week nine and 18 post treatment induction were analyzed by Olink proteomics. LOAd703 and atezolizumab did not alter levels of DC-associated proteins in plasma at nine (Fig. 1E) or 18 (data not shown) weeks post treatment initiation. Neither was an effect on circulating DC-like cells (CD14^−^HLA-DR^+^ myeloid cells) observed (Fig. 1F) but the expression of CD40 was increased on the DC-like cells nine weeks after treatment initiation (Fig. 1G). In addition, an above median frequency of CD14^−^HLA-DR^+^ cells of live cells at baseline associated with prolonged OS (Fig. 1H).

The treatment also induced expression of several genes related to tumor-associated macrophages (TAMs) and myeloid-derived suppressor cells (MDSCs) at nine (Fig. 1I, Supplementary Fig. 2A and Supplementary Table 2) and 27 weeks after treatment initiation (Supplementary Fig. 2B-C and Supplementary Table 3) with HMP of 0.00027 and 0.0012 at week nine and 27, respectively. Less impact on TAM/MDSC proteins in plasma were observed (Fig. 1J) but increased levels of circulating ARG1 were observed at week nine (Fig. 1J, *n* = 16, q-value = 0.047) which were restored to baseline at week 18 (data not shown). Circulating levels of M-MDSCs (defined as CD11b^+^CD14^+^HLA-DR^low^ cells of live cells) were not altered by the treatment (Fig. 1K) but when patients were dichotomized based on above or below median frequency of M-MDSCs of live cells at week nine, we observed an association between above median frequencies of M-MDSCs and reduced OS (Fig. 1L). Additionally, K-means clustering was exploited to test if the TAM/MDSC protein signature was associated with OS. Two clusters were generated on pre-treatment protein level (Fig. 1M) and fold change (Fig. 1N), referred to as TAM/MDSC^low^ and TAM/MDSC^high^. The TAM/MDSC^low^ clusters were associated with significantly longer OS compared with the TAM/MDSC^high^ clusters (Fig. 1O-P).

### LOAd703 and atezolizumab treatment result in elevated levels of T cell-associated genes in the tumor microenvironment

**Fig. 2:**
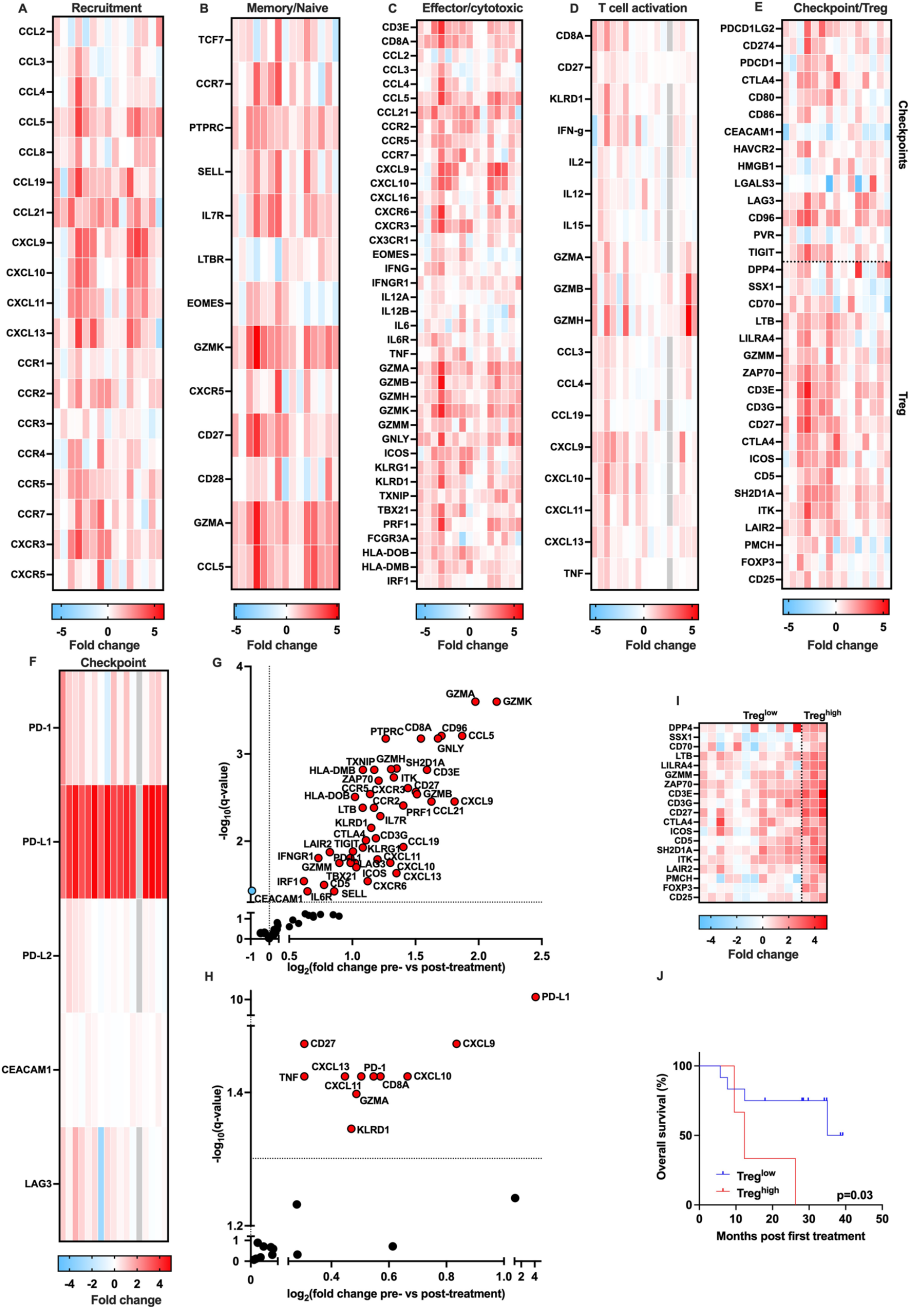
LOAd703 and atezolizumab trigger elevated levels of T cell-associated gene expression and proteins in the tumor microenvironment and plasma, respectively. Gene expression in tumor biopsies at baseline (pre) and at nine weeks post treatment induction (post) were measured using NanoString. (**A**-**C**) Fold change in gene expression between baseline and at nine weeks following treatment induction of genes associated with T cell recruitment (**A**), memory and naïve T cells (**B**), and effector and cytotoxic T cells (**C**). Protein expression in plasma was evaluated with the Olink 96 target Immuno-oncology and Oncology II proteomics panels before (pre) and at nine weeks (post) after treatment onset. (**D**) Fold change expression, post versus pre, of T cell-associated proteins. (**E**) Fold change, post versus pre, of checkpoint- (upper) and Treg- (lower) associated genes between baseline and post-treatment. (**F**) Fold change plasma expression, post versus pre, of checkpoint-associated proteins. (**G-H**) Volcano plot of T cell and checkpoint associated genes (**G**) and proteins (**H**) in the tumor microenvironment and plasma, respectively, in pre- and post-treatment (week nine) samples. In volcano plots, red indicates proteins/genes more highly and blue proteins/genes more lowly expressed post-treatment compared with pre-treatment. Statistics by paired *t* tests with correction for multiple comparison by the false discovery rate (5%) method of Benjamini Hochberg. (**I**-**J**) K-means clustering on Treg-associated gene fold change (pre- versus nine weeks after treatment initiation) was performed and heatmap shows fold change in the two clusters (**I**). (**J**) Kaplan-Meier curve of Treg^high^ and Treg^low^ clusters. Statistics were conducted by the Log rank Mantel-Cox test. Results from gene expression and protein levels are generated from 15 and 17 paired pre- and post-treatment samples, respectively

To explore if the enhanced activation of DCs triggered increased tumor infiltration of T cells, treatment induced effects on T cell recruitment-associated genes were examined. The results revealed an overall induction, both at nine (Fig. 2A) and 27 weeks (Supplementary Fig. 3A) after treatment initiation with HMP of 0.00024 and 0.00074, respectively. Genes associated with a T cell memory/naïve (Fig. 2B, HMP: 1.79 × 10^− 5^ and Supplementary Fig. 3B, HMP: 0.00046) and effector/cytotoxic (Fig. 2C, HMP: 4.92 × 10^− 5^ and Supplementary Fig. 3C, HMP: 0.00077) profile were likewise significantly induced in the TME. In addition, we observed a strong correlative effect between levels of CD8A and genes associated with recruitment of effector/cytotoxic T cells as well as biomarkers for this CD8^+^ T cell subset (Supplementary Fig. 4). The treatment also induced increased expression of T cell-related proteins in plasma at nine weeks post treatment initiation (Fig. 2D and Supplementary Table 4) with an HMP of 0.016. In agreement, levels of CD8A, CD27, IL-12, GZMH, CXCL9, CXCL10, and TNF were enhanced at 18 weeks post treatment initiation, but these inductions did not remain significant after correction for multiple comparison (Supplementary Table 5).

To explore effects on T cell suppression, markers associated with checkpoints and regulatory T cells (Treg) were analyzed. The levels of checkpoint- and Treg-associated genes in the TME (Fig. 2E, HMP checkpoints: 0.00031, HMP Treg: 0.00072 and Supplementary Fig. 3D, HMP checkpoints: 0.0026, HMP Treg: 0.0011) and PD-1 and PD-L1 in plasma (Fig. 2F and Supplementary Table 4, HMP: 9.2 × 10^− 13^) were enhanced following treatment. Significantly altered T cell and checkpoint-related genes and proteins at week nine and 27 are displayed in Fig. 2G-H, Supplementary Fig. 3E, and Supplementary Tables 2–5. Of note, post-treatment gene expression of evaluated checkpoints did however not correlate to OS in univariate Cox regression analysis. To explore a potential impact of Tregs for treatment outcome, K-means clustering on fold change of the Treg gene signature was performed which rendered a Treg^high^ and a Treg^low^ cluster (Fig. 2I). The Treg^low^ cluster associated with longer OS compared with the Treg^high^ cluster (Fig. 2J).

### Increased circulating effector memory CD8^+^ T cells post LOAd703 and atezolizumab

**Fig. 3:**
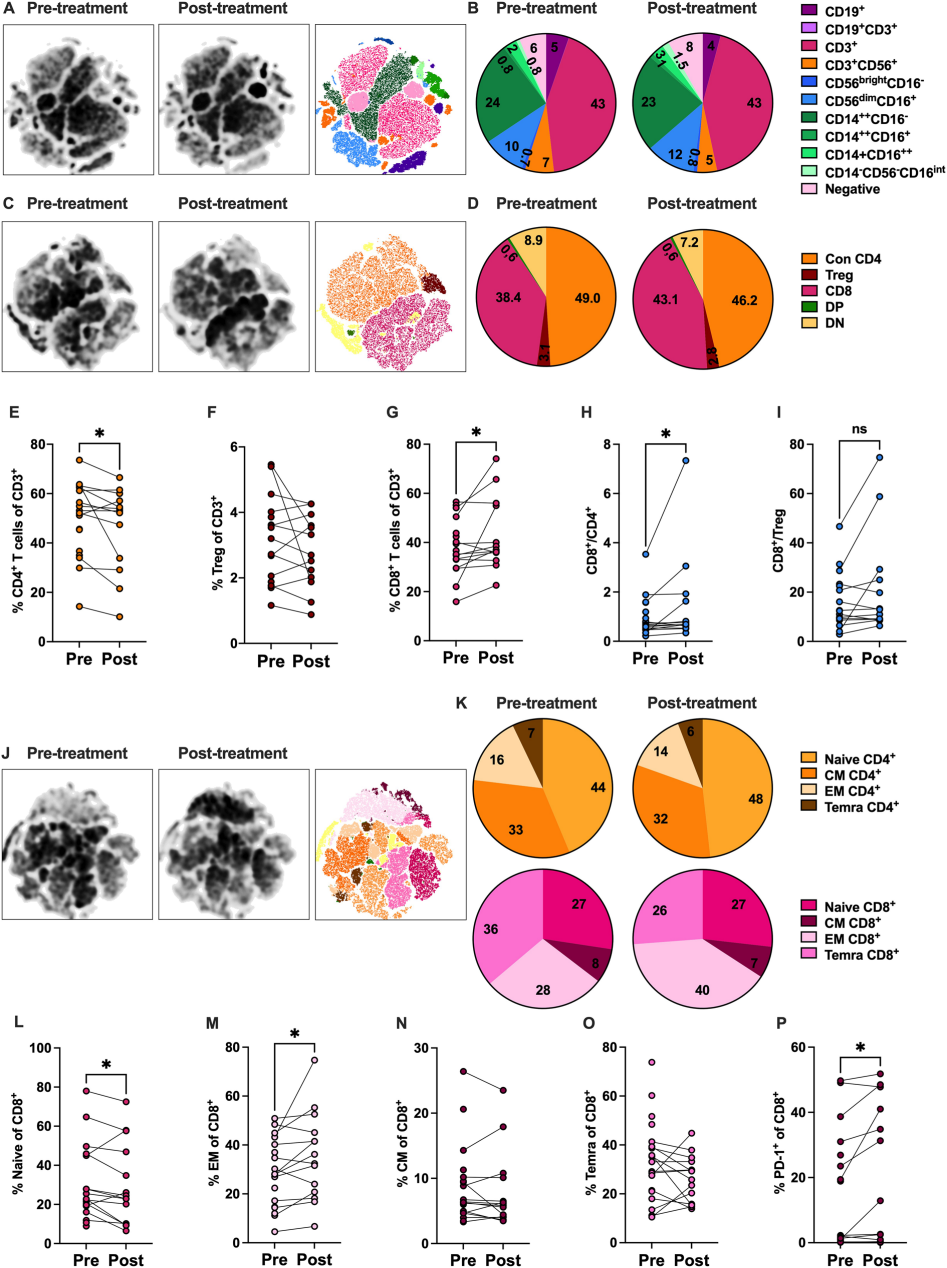
LOAd703 and atezolizumab induce increased levels of circulating effector memory CD8^+^T cells in malignant melanoma patients. Peripheral blood mononuclear cell (PBMC) samples were collected before treatment and at nine weeks after treatment onset and analyzed by flow cytometry for frequencies and activation state of immune populations. (**A**) Unsupervised clustering of 10 000 live cells from each sample based on expression of CD16, CD56, CD3, CD19, and CD14 were performed using Phenograph. Twentynine clusters were identified which were manually pooled into eleven immune cell populations displayed as tSNE density plots pre- and post-treatment and combined with color coding. (**B**) Frequencies of immune cell populations depicted in A pre-and post-treatment. (**C**) Unsupervised clustering of 5000 live CD3^+^ cells from each sample based on expression of CD4, CD8, CD25, and CD127 were performed using Phenograph. Thirty-one clusters were identified which were manually pooled into five T cell populations displayed as tSNE density plots pre- and post-treatment and combined with color coding. (**D**) Frequencies of T cell populations depicted in B pre-and post-treatment. (**E-I**) Frequencies of conventional CD4^+^ T cells (**E**), Treg (**F**), CD8^+^ T cells (**G**), CD8^+^/CD4^+^ (**H**), and CD8^+^/Treg (**I**) ratios identified by flow cytometry in pre- and post-treatment PBMC samples. (**J**) 5000 live CD3^+^ cells from each sample were concatenated and cells were defined as naïve, central memory (CM), effector memory (EM), and terminally differentiated effector memory (Temra) T cell subsets based on CCR7 and CD45RA expression. Data is presented as tSNE density plots pre- and post-treatment and combined with color coding. (**K**) Frequencies of CD4^+^ (upper panels, orange) and CD8^+^ (lower panels, pink) T cell memory populations depicted in J pre- and post-treatment. (**L-O**) Frequencies of naïve (**L**), effector memory (EM) (**M**), central memory (CM) (**N**), and terminally differentiated EM (Temra) (**O**) CD8^+^ T cells in pre- and post-treatment PBMC samples. (**P**) Frequencies of PD-1^+^ CD8^+^ T cells in pre- and post-treatment PBMC samples. Statistics were conducted by the Wilcoxon test. *p < 0.05, ns=non-significant

Next, treatment induced effects on circulating leukocytes were explored. Unsupervised cluster-based analysis of flow cytometric data indicated no alterations in the assessed immune populations following treatment (Fig. 3A-B). A more detailed analysis of T cell subtypes, however, revealed that patients presented with reduced frequencies of conventional CD4^+^ T cells post-treatment (Fig. 3C-E) with a similar trend observed for Tregs (Fig. 3C-D and F). In-depth analysis, with a traditional gating strategy, in fact revealed a significant reduction also of Tregs (Supplementary Fig. 5A-B). In contrast, frequencies of CD8^+^ T cells were elevated in post-treatment samples (Fig. 3C-D and G) resulting in an increased CD8^+^/CD4^+^ T cell ration post-treatment (Fig. 3H) with a similar trend for the CD8^+^ T cell/Treg ratio (Fig. 3I). Again, traditional gating resulted in a significant increased CD8^+^ T cell/Treg ratio (Supplementary Fig. 5C). No significant changes were observed for CD4^+^ T cell memory subsets (Fig. 3J-K), but we noted a reduction in naïve CD8^+^ T cell frequencies (Fig. 3K-L) which coincided with an induction of effector memory (EM) CD8^+^ T cells (Fig. 3M) succeeding treatment. Frequencies of central memory (CM) and terminally differentiated EM (Temra) CD8^+^ T cells were not affected by the therapy (Fig. 3N-O) but increased expression of PD-1 on CD8^+^ T cells was observed (Fig. 3P).

### LOAd703 and atezolizumab promote increased levels of NK cell-associated biomarkers

**Fig. 4:**
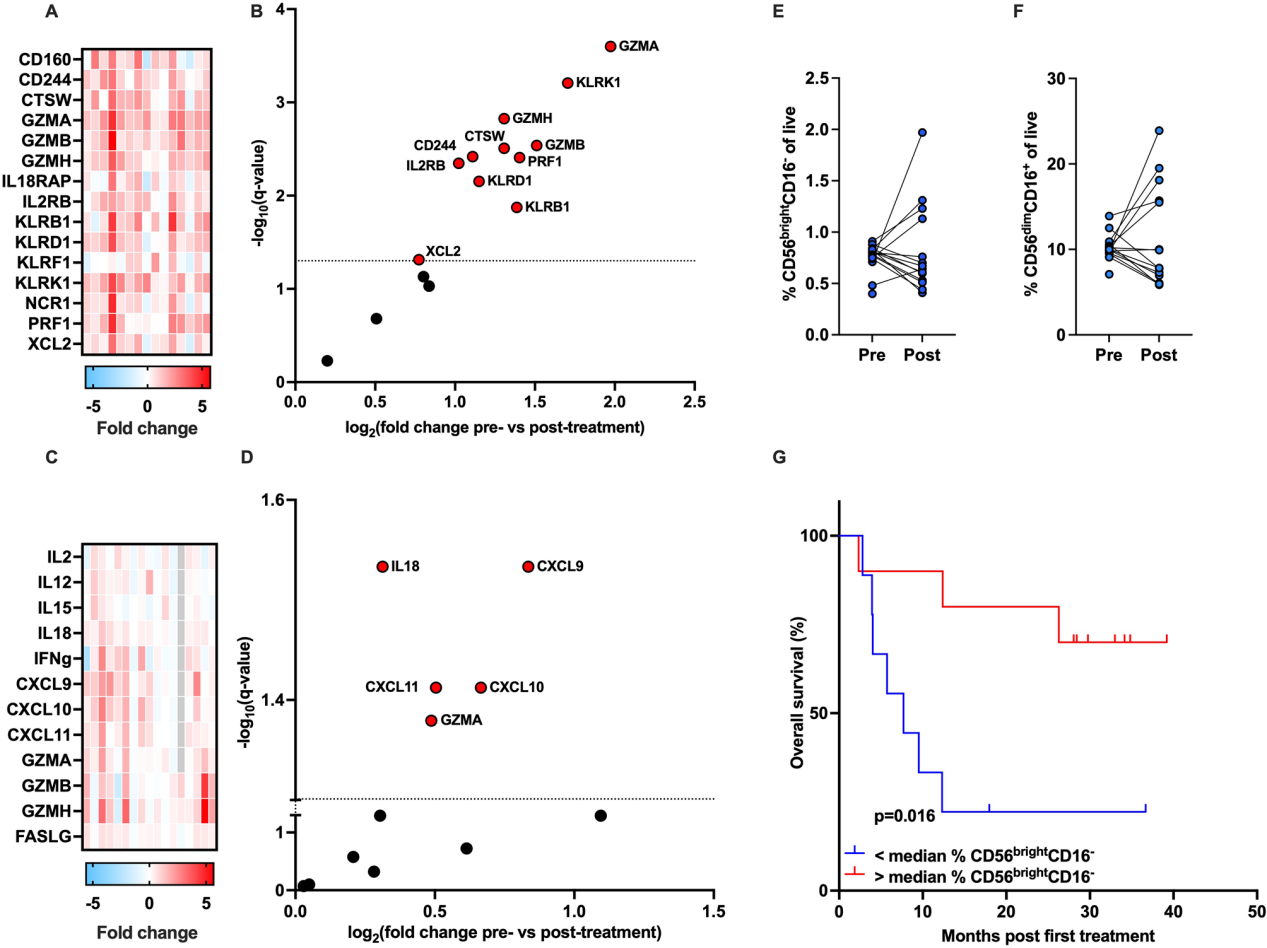
LOAd703 and atezolizumab promote increased levels of NK cell-associated gene expression in the tumor microenvironment and proteins in plasma. NK cell-associated gene expression in tumor biopsies and protein expression in plasma was measured at baseline (pre) and at nine weeks post treatment induction (post) using NanoString and the Olink 96 target Immuno-oncology and Oncology II proteomics panels. (**A**) Fold change expression of NK cell-associated genes in the tumor microenvironment, pre versus post treatment. (**B**) Volcano plots of NK cell-associated genes in the tumor microenvironment, pre versus post treatment. (**C**) Fold change expression of NK cell-associated proteins in plasma, pre versus post treatment. (**D**) Volcano plots of NK cell-associated proteins in plasma, pre versus post treatment. In volcano plots, red indicate genes/proteins more highly expressed post-treatment compared with pre-treatment. Statistics by paired t tests with correction for multiple comparison by the false discovery rate (5%) method of Benjamini Hochberg. (**E**-**F**) Alterations of NK cells among peripheral blood mononuclear cells were assessed at baseline (pre) and at nine weeks (post) after treatment initiation by flow cytometry. Pre- and post-treatment frequencies of CD56^brigh^CD16^−^ (**E**), and CD56^dim^CD16^+^ (**F**) NK cells of live cells. (**G**) Kaplan-Meier curve of patients based on an above or below median frequency of CD56^bright^CD16^−^ cells of live cells at baseline. Statistics were conducted by the log-rank Mantel Cox test. Results from gene expression and protein levels are generated from 15 and 17 paired pre- and post-treatment samples, respectively

Evaluation of NK cell-associated genes from the transcriptomic tumor analysis revealed increased expression of several NK cell-associated genes nine (Fig. 4A-B and Supplementary Table 2, HMP: 3.89 × 10^− 5^) and 27 (Supplementary Fig. 6A-B and Supplementary Table 3, HMP: 0.00041) weeks after treatment initiation. Induction of NK cell-associated proteins in plasma was also noted at nine weeks post treatment initiation (Fig. 4C-D and Supplementary Table 6, HMP: 0.015). Cluster-based analysis of flow cytometric data detected cytokine producing CD56^brigh^CD16^−^ and cytotoxic CD56^dim^CD16^+^ NK cells in pre- and post-treatment PBMC samples (Fig. 3A-B). However, LOAd703 and atezolizumab did not result in altered frequencies of neither NK cell subtype (Fig. 4E-F). Nevertheless, patients having an above median frequency of CD56^bright^CD16^−^ NK cells of live cells at treatment onset displayed association with longer OS compared with patients having below median frequency of CD56^bright^CD16^−^ NK cells (Fig. 4G).

### CD8A represent a central node in the intratumoral genetic signature of LOAd703 and atezolizumab treated patients

**Fig. 5:**
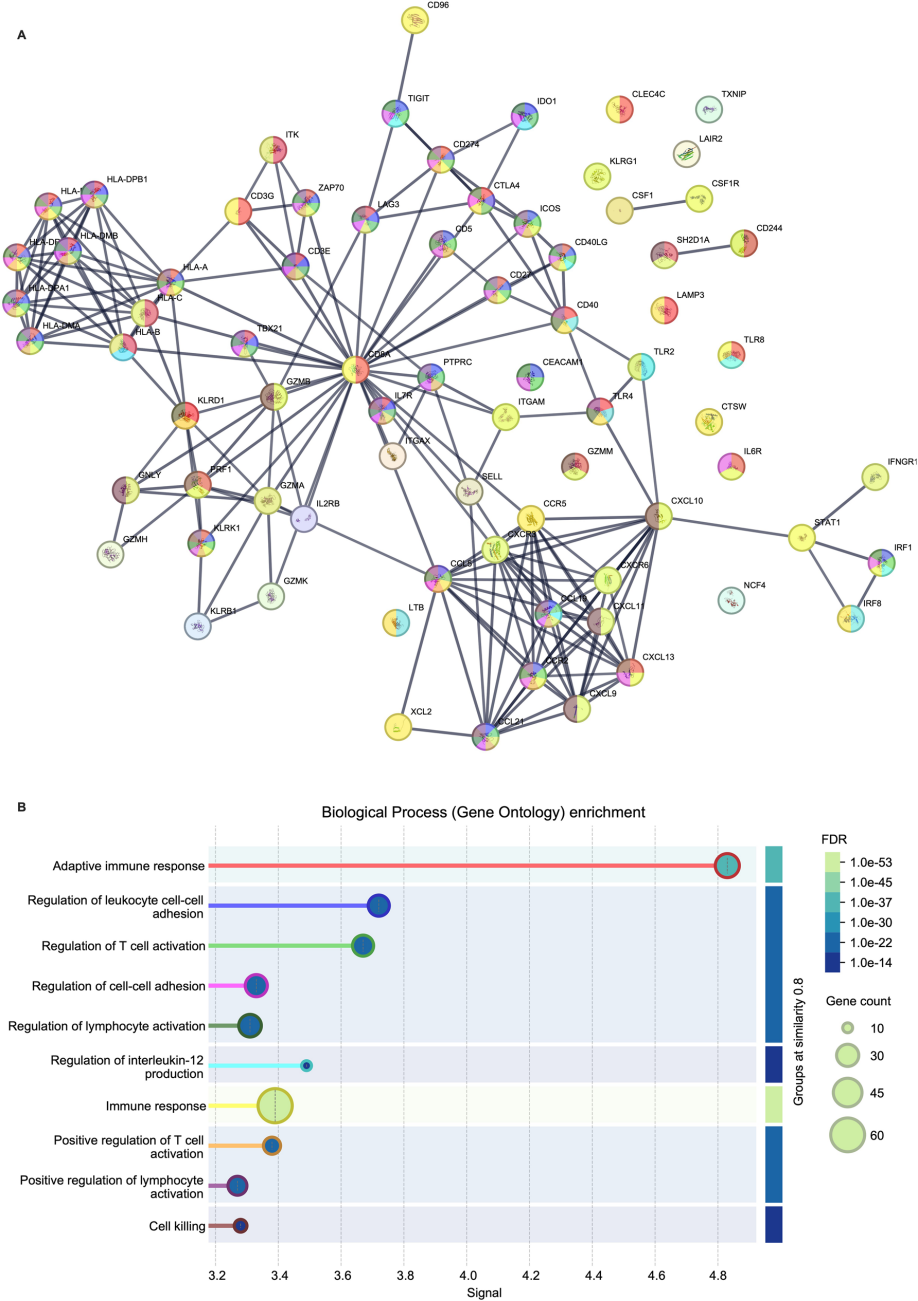
LOAd703 and atezolizumab induce genes are involved in regulating T cell activation. (**A**) Pathway analysis of encoded proteins of significantly altered dendritic cell (DC), antigen presenting, tertiary lymphoid structure, myeloid-derived suppressor cell, tumor associated macrophage, T cell, checkpoint, and NK cell associated genes. (**B**) Gene set enrichment analysis including the top ten hits based on signal (weighted harmonic mean between the ratio of observed versus expected genes in the respective pathway and the false discovery rate). Proteins in **A** are color-coded based on the gene set enrichment analysis in **B**. Pathway analysis was conducted using the STRING database

To further elaborate on treatment effects in the TME, the assessed immune cell prototypic genes significantly altered by the therapy were included in a gene ontology (GO) enrichment analysis using the STRING database (RRID: SCR_005223). The results identified CD8A as a central node strongly related to expression of a cluster of HLA molecules, a cluster of cytokines and chemokines, and markers associated with positive T cell activation and cell killing (Fig. 5A-B). While genes associated with DCs, antigen presentation, T cells, and NK cells displayed an associative relation, TAM/MDSC-prototypic genes appeared to relate to these genes to a lesser extent.

### Genes associated with defense against viruses negatively associated with OS while genes associated with T cell fitness and melanoma antigens associated with longer OS

**Fig. 6:**
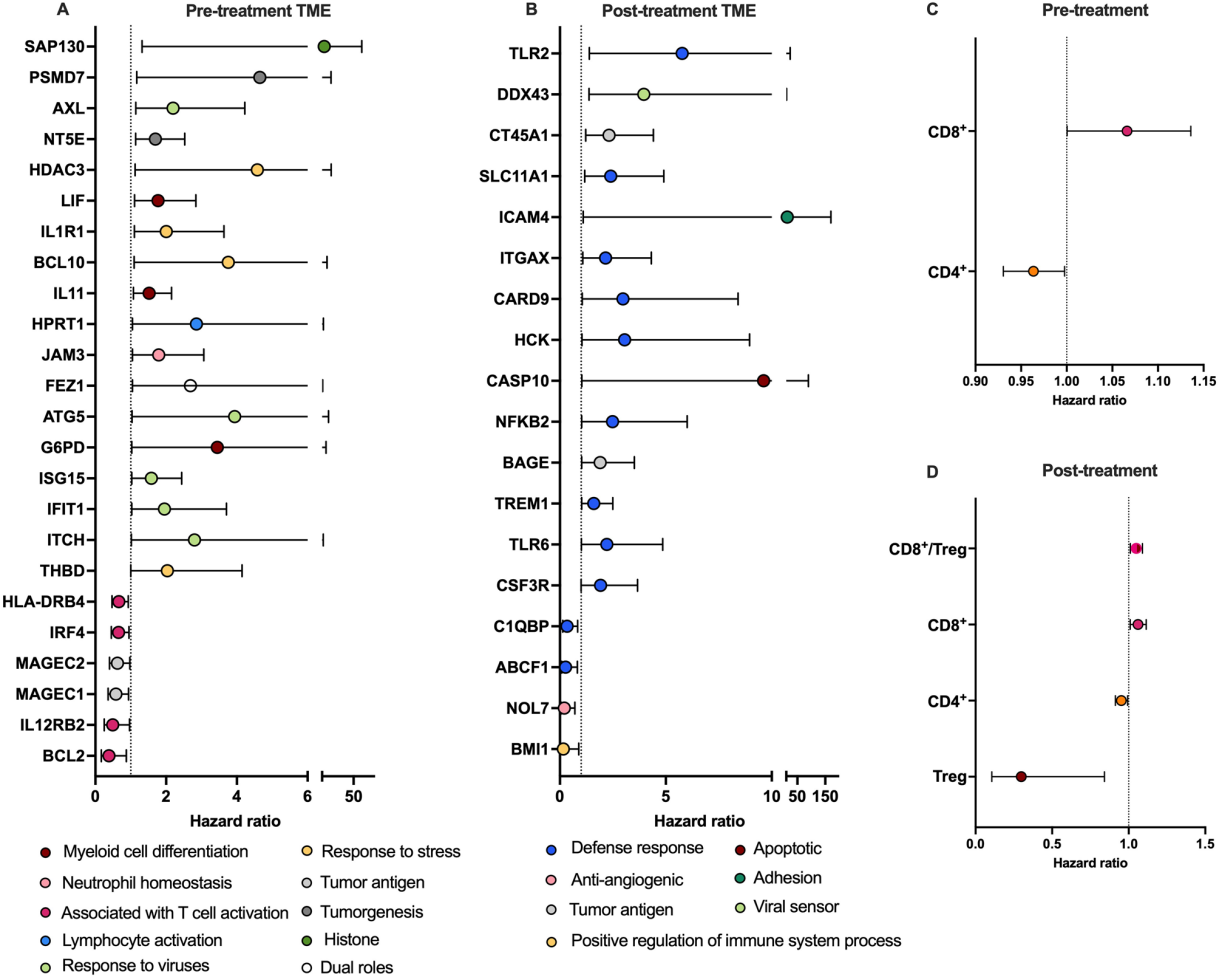
Genes associated with T cell activation and/or fitness and circulating levels of T cell subsets significantly associated with overall survival after LOAd703 and atezolizumab treatment. Forest plots were generated by univariate Cox regression analysis of expression level of all 770 genes included in the NanoString panel (**A**-**B**) and frequencies of circulating leukocytes (**C**-**D**) at baseline (**A** and **C**) and nine weeks following treatment induction (**B** and **D**). Gene expression (**A**-**B**) or frequencies of leukocytes (**C**-**D**) that significantly predicted longer overall survival (*p* < 0.05 and confidential interval for hazard ratio < 1) or shorter (*p* < 0.05 and confidential interval for hazard ratio > 1) overall survival are included in the forest plots. Genes in **A** and **B** are color-coded based on gene set enrichment analysis (supplementary Figs. 7 and 8) and literature review

We next aimed to explore the associations between gene expressions in the TME and OS. Univariate Cox regression analysis of all 770 genes included in the TME transcriptome analysis was performed. Genes that significantly associated with OS pre- or post-treatment are displayed in Fig. 6A-B, respectively. Pathway and GO enrichment analyses of pre- (Supplementary Fig. 7A-B) and post-treatment (Supplementary Fig. 8A-B) gene expression correlative with OS were performed. Based on the GO enrichment analysis and literature review, the genes were defined and color-coded by function (Fig. 6A-B). The results suggest that high pre-treatment expression of the melanoma antigens, MAGE-C1 and -C2, as well as high expression of IRF4 and HLA-DRB4, genes involved in T cell activation, and anti-apoptotic BCL2 associate with longer OS. On the contrary, enhanced pre-treatment levels of genes associated with tumorigenesis and innate immune responses, many associated with myeloid cells or Th2 polarization of T cells, and response to stress or viral clearance were associated with shorter OS (Fig. 6A and Supplementary Fig. 7A-B). High post-treatment levels of BMI1 and C1QBP, genes previously implicated in T cell fitness and maturation [14, 15], anti-angiogenic NOL7, and immunoregulating ABCF1 associated with prolonged OS while enhanced expression of genes associated with innate immune responses, in particular TLR6 and TLR2 signaling, macrophage activation, IL-6 signaling, tumor antigens, and defense response, were associated with shorter OS (Fig. 6B and Supplementary Fig. 8A-B). Of note, none of these associations remained significant in multivariate Cox regression analysis, possibly explained by the high correlative expression between these genes.

Univariate Cox regression analysis was also conducted for peripheral frequencies of immune cell populations. High baseline and post-treatment frequencies of CD4^+^ T cells was associated with longer OS. Similarly, high frequencies of Tregs at nine weeks after treatment induction were associated with longer OS. In addition, low baseline and post-treatment frequencies of CD8^+^ and a low CD8^+^/Treg ratio at nine weeks post treatment initiation associated with shorter OS (Fig. 6C-D). Again, none of these associations remained significant in multivariate Cox regression analysis. Of note, while frequencies of M-MDSCs nine weeks post treatment onset and pre-treatment frequencies of CD14^−^HLA-DR^+^ DC-like cells associated with OS in Kaplan-Meier analysis we did not observe significant effects of their frequencies in Cox regression analysis although the same trends were observed (hazard ratio = 1.15 and *p* = 0.15 for pre-treatment frequencies of MDSCs and hazard ratio 0.86 and *p* = 0.12 for DC-like cells).

### Correlation between plasma and tumor microenvironment biomarker expression

We next correlated fold change (week nine versus baseline) of plasma proteins with gene expression in the TME. Supplementary Fig. 9 displays Pearson r (Supplementary Fig. 9A) and accompanying p-value (Supplementary Fig. 9B) of all plasma proteins significantly correlated with one or more genes in the TME. Of interested, proteins associated with immunosuppression, such as PD-L1, CEACAM1, and NOS2, were among the proteins correlating with most genes in the TME. Additionally, many of the markers associated with immunosuppression displayed negative association with gene expression in the TME.

## Discussion

Resistance to ICIs in MM patients remains a significant clinical hurdle which warrants the urgent need for treatments that (re)-introduce ICI sensitivity [2]. An immune excluded or desert TME often characterizes ICI resistance and means to boost immune cell infiltration has been postulated to improve responses. We recently reported promising clinical outcomes from the LOKON003 phase I/II clinical trial where ICI resistant, stage IV, MM patients received combined treatment with LOAd703 and atezolizumab. The treatment was largely well-tolerated with an encouraging median overall survival of 19.3 months [16]. Using cellular, proteomic, and transcriptomic analysis of blood and tumor biopsies from patients included in the LOKON003 trial, we herein report that LOAd703 and atezolizumab reprogram the immunological signatures of ICI resistant MM to a signature typical for ICI responsiveness [17].

Although treated patients had metastatic disease, LOAd703 was locally administered into a selected tumor lesion. A previous report suggest that repeated systemic administration of an adenovirus-based therapy elevate levels of anti-adenoviral antibodies in patients [18] potentially limiting treatment efficacy. Local administration of LOAd703 was hypothesized to circumvent circulating anti-viral antibodies and thus limit the risk of adverse events and reduced efficacy. The local administration route may, however, raise concerns in regard to systemic treatment effects and the capacity to evoke long-term anti-tumor immune memory. We noted induction of NFκB1A and STAT2, genes associated with anti-adenoviral immune response, in the TME [16] and observed an increment in plasma anti-drug antibodies (Naseri et al. submitted manuscript, 2025) following treatment. Tumor microenvironment expression of genes involved in response to and clearance of viruses also correlated with reduced OS. Although anti-viral immunity may negatively impact on treatment response, the results also revealed several systemic immunological effects of the treatment, including signs of induced immune memory, suggesting that local LOAd703 administration augment systemic immune activation.

The mechanistic action of LOAd703 is hypothesized to strongly relay on induced activation of DCs. We have previously shown improved DC maturation in vitro following infection with LOAd703 or co-culture with LOAd703-infected melanoma cells [10, 12]. In agreement, several markers associated with DCs and antigen presentation were enhanced in the TME and elevated expression of CD40 on peripheral DC-like cells was noted post-treatment. We also observed increased expression of both CD40L and CD40 in the TME. Of note, encouraging results were recently reported for combined anti-PD-1 and agonistic CD40 antibodies in ICI resistant MM patients [19]. Preclinical work suggests that high CD40 expression on DCs favors Th1 type immune response [20]. Th1 cytokines are further known to amplify MHC I and II expression. We observed significantly higher MHC I and II expression in the TME post treatment. Downregulation of MHC I on tumor cells is a well-documented cause of ICI resistance. Since our transcriptomic analysis was performed on bulk mRNA from tumor biopsies, we cannot deduce any potential impact of LOAd703 and atezolizumab on tumor versus immune cell MHC expression. However, in support of a favorable correlation between levels of MHC I and clinical benefit following ICI treatment, downregulation of MHC I in MM is associated with reduced ICI response [5]. Tumor cell expression of MHC II is also positively correlated with response to ICI in MM and non-small cell lung cancer (NSCLC) [21]. In coherence, we observed a positive correlation between OS and pre-treatment expression of HLA-DRB4.

While increased MHC I and II expression in the TME may stem from enhanced expression on tumor cells, several additional markers associated with DCs and antigen-presentation were elevated by the treatment regimen suggesting a significant impact of the treatment on DCs. These include the type 1 conventional DC transcription factor IRF8 and the DC3 marker LAMP3. In NSCLC, high intratumoral IRF8 levels enhance anti-tumor immune response [22] while infiltration of LAMP^+^ DCs has been reported prognostic in CMM [23]. Several of the DC-associated markers in the TME are also enhanced in advanced cancer patients receiving LOAd703 without supplementary anti-PD-L1 treatment (Wenthe et al. submitted manuscript, 2025). Of note, a de-differentiated phenotype and reduced DC infiltration in tumors are associated with lack of response to ICIs [24]. To our knowledge, induction of DC-associated biomarkers in ICI resistant MM patients has not previously been observed following ICI monotreatment. Collectively the results thus suggest that the treatment regimen, significantly contributed by LOAd703, triggers activation of DCs, which may aid to overcome ICI resistance.

Increased activation of intratumoral DCs may evoke formation of TLS. Tertiary lymphoid structures positively impact on OS in cancer [25] and are associated with favorable responses to ICIs in MM [26]. Pre-clinical work suggests that induced TLS formation represses MM growth [27] and heightens ICI treatment efficacy [28]. We observed increased expression of TLS-associated markers following LOAd703 and atezolizumab treatment in previously ICI resistant patients. The results thus suggest that the treatment regimen is able to provoke a TLS profile characteristic of ICI responsiveness.

The pathway analysis of genes significantly altered by LOAd703 and atezolizumab revealed a relation between many of the DC- and antigen presentation-associated genes and T cell signature genes. The significant induction of the T cell recruitment gene panel also denotes an improved T cell recruitment profile. Many of these genes strongly correlated with TME expression of CD8A, indicating their supportive effect on T cell influx. In agreement, following treatment, patients presented with enhanced levels of several T cell associated genes, including markers for memory, naïve, cytotoxic, and effector molecules. Similar effects were also noted in the LOKON002 trial (Wenthe et al. submitted manuscript, 2025) where advanced solid cancer patients were treated with LOAd703 in combination with chemotherapy that otherwise reduces these markers [29]. Thus, indicating that the effects are predominantly mediated by LOAd703. Collectively, these results imply that patients harbor improved T cell tumor infiltration following LOAd703 and atezolizumab treatment. 

Previous publications suggest encouraging impact of T cell recruitment and influx on response to ICIs. Analysis of The Cancer Genome Atlas, including over 9000 tumors, revealed a positive correlation between expression of CD8A and CLL5 and CXCL9 in the TME which was associated with prolonged response to ICIs [30]. In accordance, CCL4, CCL5, CXCL9, CXCL10, and CXCL11 were more highly expressed in pre-treatment tumor biopsies from MM patients responsive to ICIs compared with levels in non-responding patients and these levels also correlated with higher tumor lymphocyte infiltration score [31]. The observed T cell effects in the TME following LOAd703 and atezolizumab treatment have thus previously been reported associative with ICI response. Again, these results suggest a reprogramming of the TME immune signature of ICI resistant patients to that of responding patients by the treatment regimen.

High pre-treatment expression of the melanoma antigens, MAGE-C1 and -C2, genes involved in T cell activation, IRF4 and HLA-DRB4, and anti-apoptotic BCL2 was associated with longer OS. The positive impact of mutational burden on response to ICI is well-established owing to the increased likelihood of achieving T cell activation in the presence of neoantigens and/or cancer testis antigens (CTAs) [32]. Expression of the CTAs MAGE-A3 and -A4 are associated with ICI response in MM [33]. While anti-MAGE-C specific cytotoxic lymphocytes have been identified in MM [34], the potential of MAGE-C to impact ICI response remains to be elucidated. To our knowledge, we are the first to show an association between baseline TME MAGE-C expression and longer OS following immunotherapeutic treatment. The IRF4 transcription factor has multifarious roles in immune cells. Although IRF4 is acknowledged as a critical transcription factor for Th2 activation, IRF4 has also been implicated in clonal expansion and maintenance of effector functions of antigen-specific CD8^+^ T cells [35]. High tumor cell expression of BCL2 may provide tumor cells with increased resistance against apoptosis and thus positively impact on tumorigenesis. However, BCL2 has also been reported elevated in CD8^+^ memory T cells compared with naïve CD8^+^ T cells [36]. Overexpression of BCL2 in tumor-specific T cells reportedly improve the potency of an adoptive T cell therapy in a murine MM model [37]. High post-treatment expressions of C1QBP and BMI1 were also associated with longer OS. C1QBP and BMI1 have been implicated in tumor progression but also in regulation of anti-tumor immunity [14, 15]. Interestingly, C1QBP T cell deficiency renders reduced BCL2 expression, thus accelerating T cell apoptosis and triggering aggravated tumor infiltrating lymphocyte exhaustion [15]. BMI1 supports long-lived memory T cells while weakening short-lived effector T cells [14]. In hepatocellular carcinoma (HCC), elevated BMI1 expression of circulating CD8^+^ T cell correlated with improved response to ICIs [14]. Collectively, genes associated with T cell fitness and genes implicated in CD8^+^ memory differentiation positively correlated to OS following LOAd703 and atezolizumab treatment.

Previous reports suggest that advanced MM patients responsive to ICIs mount increased proliferation of circulating CD8^+^ T cells [38] with reduced levels of naïve CD8^+^ T cells [39] following treatment while induction of Tregs has been reported for non-responding patients [38]. In HCC, treatment with atezolizumab triggers elevated levels of CD8^+^PD-1^+^ lymphocytes in responding patients while an opposite trend was observed in non-responding patients [40]. Previous data thus support divergent peripheral immune responses to ICIs between clinically responding and non-responding patients. Our data indicates that combined treatment with LOAd703 and atezolizumab increased the frequency of CD8^+^ T cells with a parallel reduction of CD4^+^ cells and Tregs. We also observed induced PD-1 expression on CD8^+^ T cells and EM CD8^+^ T cell polarization. The results thus suggest that combined LOAd703 and atezolizumab therapy triggers a systemic immune profile in ICI resistant patients that resembles what has previously been reported for ICI responsive patients. In addition, the results indicate that supplementary LOAd703 treatment to anti-PD-L1 therapy aids in reprograming T cell immunity to overcome mechanisms underlying ICI resistance.

Low circulating levels of CD8^+^ T cells and high frequencies of CD4^+^ T cells before treatment onset were associated with longer OS. The same applies to high frequencies of CD4^+^ T cells and Tregs, low CD8^+^ T cell frequencies and a low CD8^+^/Treg ratio post treatment. Hence, patients in which we observed the most vigorous systemic immune effects comprise patients who experienced shorter survival. High frequencies of CD8^+^ T cells are generally regarded as a positive prognostic marker in cancer. An association between low circulating CD8^+^ T cell frequencies and longer OS may thus seem counterintuitive given their well-documented role in anti-tumor immunity. However, reduced levels of circulating CD8^+^ T cells may reflect an increased CD8^+^ T cell recruitment from the periphery to the TME. It can be postulated that therapies that benefit from increased lymphocyte infiltration of tumors, such as ICIs and LOAd703, may profit from draining of anti-tumor lymphocytes in circulation. In support of this assumption, LOAd703 and atezolizumab triggered elevated levels of several chemokines and chemokine receptors in the TME known to be involved in tumor infiltration of T cells. In addition, we observed that patients having high frequencies of circulating Tregs displayed longer OS. On the contrary, patients with high expression of Treg-associated genes in the TME had adverse prognosis suggesting that the immunological profile in periphery may not reflect that of the TME. It is thus plausible to speculate that patients with lower circulating levels of CD8^+^ T cells correspond to patients in which a more robust influx of CD8^+^ T cells in the TME have occurred explaining the negative association between circulating CD8^+^ T cell levels and OS.

Circulating levels of NK cells were not affected by the therapy, but we did note an association between clinical benefit and an above median frequency of CD56^bright^CD16^−^ NK cells of live cells at treatment onset. CD56^bright^CD16^−^PD-1^+^ NK cells have previously been shown to associate with good response to ICIs in NSCLC [41]. Increased infiltration of CD56^dim^CD16^−^ NK cells in the TME is also associated with beneficial outcome in MM [42]. CD16^−^ NK cells are generally regarded as less cytotoxic, compared with CD16^+^ NK cells, but with increased cytokine producing ability. Thus, their anti-tumor effector function likely lies within their cytokine, chemokine, and growth factor producing ability. The mechanistic action of LOAd703 conveys on its ability to mount innate and adoptive anti-tumor immune activation. These processes relay on a favorable cytokine milieu. Several reports suggest that NK cell-derived cytokines contribute in promoting DC activation [43], a pivotal process for LOAd703 potency. Combined treatment of tumor-bearing mice with IL-18 and anti-PD-1 was observed to activate NK cells in the TME which resulted in IL-12-dependent recruitment of type 1 conventional DCs [44]. Of note, we observed significantly higher levels of IL-18 in plasma post LOAd703 and atezolizumab treatment and enhanced expression of several NK cell- and DC-associated markers in the TME. Further, preexisting NK cells were required for potency of a DC vaccine strategy in murine MM [45]. A DC vaccine was recently also evaluated in a phase I clinical trial in MM. CD56^dim^CD16^−^ NK cells were found to be the most dominant NK cell subset in the TME and a trend for an association between increased CD56^dim^CD16^−^ gene profile in the TME and better clinical outcome was observed [42]. In addition, preclinical work reports NK cell-dependent synergistic effects between an adenoviral vector expressing IL-12 and anti-4-1BB monoclonal antibodies. Adenovirus-induced IL-12 was found to drive NK cell-mediated IFN-γ production resulting in activation of 4-1BB^+^ DCs and increased tumor infiltration of DCs with elevated expression of MHC class II [46]. CD56^bright^CD16^−^ NK cells may thus provide an increased capacity to mount DC-supporting cytokine production following treatment with LOAd703.

We observed significantly elevated levels of soluble PD-L1 in plasma post treatment. The induction of PD-L1 is likely derived from the atezolizumab treatment as such robust effect of soluble PD-L1 in plasma was not observed in advanced staged cancer patients treated with LOAd703 in combination with chemotherapy (Wenthe et al. submitted manuscript, 2025). In contrast, we noted strong induction of soluble PD-L1 in pancreatic cancer patients after atezolizumab and chemotherapy treatment (manuscript in preparation). Furthermore, a 50-fold increment of soluble PD-L1 levels in serum after monotherapy with atezolizumab has been reported [47]. Jointly these results imply that the enhanced levels of soluble PD-L1 post treatment is likely attributed to atezolizumab, possibly due to increased half-life owing to antibody-PD-L1 complexes, increased release of soluble PD-L1 from cells, or both. We do however also note enhanced gene expression of checkpoints, including LAG-3, TIGIT, and CTLA-4, in the TME post LOAd703 and atezolizumab treatment. Induce expression of alternative immune checkpoints is a well-recognized response to ICI that may drive treatment resistance [5, 7] but is also associated with an inflamed hot TME. Early induction of checkpoints following ICI has been proposed to associate with response to immunotherapies while a later induction may associate with exploitation of alternative immune checkpoints and emerging treatment resistance. We do not see any association between post-treatment gene expression of any assessed checkpoint in the TME and OS. However, additional clinical trials are warranted to evaluate the kinetics of altered checkpoint expression, its association with response to treatment, and a potential benefit of combining LOAd703 with a combination of ICIs to counteract the hypothetical negative effects of induced expression of checkpoints post treatment.

LOAd703 and atezolizumab did not alter systemic levels of MDSCs but we noted significant induction of ARG1 in plasma and observed elevations of several MDSC- and TAM-associated genes in the TME post treatment. High frequencies of MDSCs are known to impede response to ICIs in MM [8] also when ICIs are combined with the DC targeting therapy GVAX [48]. In agreement, we observed an association between shorter OS in patients with high levels of MDSC-associated proteins in plasma at treatment onset and high circulating frequencies of MDSCs following treatment. The same trend, although not reaching statistical significance was observed in univariant Cox regression analysis. High pre- and post-treatment TME expression of genes associated with myeloid cell differentiation and neutrophils, many of which have also been implicated to promote expansion and/or immunosuppression of MDSCs and TAMs, including CSF3R [49], TREM1 [50], and increased IL-6 signaling [51] were also associated with shorter OS. Conclusively, these results imply that suppressive myeloid cells negatively associate with response to LOAd703 and atezolizumab and suggest that a TAM/MDSC-targeting treatment may further contribute to overcome ICI resistance.

## Conclusion

We acknowledge that this trial has several limitations. First, the trial did not include a control arm and additional randomized trials are warranted to decipher the potential benefit of LOAd703 and atezolizumab to overcome ICI resistance in melanoma. Second, the results are based on a small sample size of patients and post-hoc tests. Third, bulk tumor transcriptomic analysis was performed. Nevertheless, the herein presented results suggest that treatment with LOAd703 and atezolizumab renders a local and systemic immune signature in ICI resistant MM patients resembling what has previously been reported correlative with ICI response. This includes an increased TME expression of DC-associated biomarkers, an enhanced level of circulating CD40^+^ DC-like cells, an increase in biomarkers associated with T cell infiltration and T cells in the TME, elevation of circulating CD8^+^ T cells with an EM phenotype, and reduced circulating levels of CD4^+^ T cells including Tregs. Collectively these results suggest that LOAd703 promote reprograming of the immune landscape to overcome mechanisms underlaying ICI resistance in MM. Modulating the TME to introduce ICI sensitivity using an off-the-shelf product allows for immediate treatment of patients and provides a promising tool to increase responses to immunotherapies in cancer. Additional clinical trials assessing the efficacy of LOAd703 and ICI in MM are warranted.

## Supplementary Information

Below is the link to the electronic supplementary material.

Supplementary Material 1: Document S1. Figure S1-S9, Table S1-S9. Document S2. Related manuscript by Omid et al. entitled “LOAd703-Induced Tumor Microenvironment Gene Engineering in Combination with Atezolizumab in Metastatic Malignant Melanoma – A phase I/II Trial”. The manuscript present clinical results from the LOKON003 trial and accepted for publication in *Nature Communications*

## Data Availability

Requests for further information and resources should be directed to the corresponding author, Angelica Loskog, Department of Immunology, Genetics and Pathology, Uppsala University. Dag Hammarskjöldsväg 20, 751 85 Uppsala. Email: angelica.loskog@igp.uu.se. LOAd703 sharing will be considered by the provider Lokon Pharma on a case-by-case basis. Requests by academic study groups for deidentified patient data with the intent to achieve aims of the original proposal can be forwarded to the corresponding author. Data code will be provided on request. Data is provided within the manuscript or supplementary information files.

## Abbreviations

4-1BBL: 4-1BB ligand
CD: Cluster of differentiation
CD40L: CD40 ligand
CM: Central memory
CMM: Cutaneous malignant melanoma
CTA: Cancer testis antigen
DC: Dendritic cell
EM: Effector memory
FDR: False discovery rate
GO: Gene ontology
HCC: Hepatocellular carcinoma
HMP: Harmonic mean p-value
ICI: Immune checkpoint inhibitor
MDSC: Myeloid-derived suppressor cell
MM: Malignant melanoma
NK: Natural killer
NSCLC: Non-small cell lung cancer
OS: Overall survival
PBMC: Peripheral blood mononuclear cell
TAM: Tumor associated macrophage
Temra: Terminally differentiated effector memory
TLS: Tertiary lymphoid structure
TME: Tumor microenvironment
