# Supplementary material for: Reprogramming myeloid cells and restoring T cell fitness in checkpoint inhibitor resistant melanoma patients
Source: Biomark Res. 2026 Mar 27;14:38. doi: 10.1186/s40364-026-00917-z (PMC13063715; doi:10.1186/s40364-026-00917-z)
Supplement: Supplementary file 1 — Supplementary Material 1: Document S1. Figure S1-S9, Table S1-S9. Document S2. Related manuscript by Omid et al. entitled “LOAd703-Induced Tumor Microenvironment Gene Engineering in Combination with Atezolizumab in Metastatic Malignant Melanoma – A phase I/II Trial”. The manuscript present clinical results from the LOKON003 trial and accepted for publication in Nature Communications [file 40364_2026_917_MOESM1_ESM.pdf]

Document S1: Supplementary Information

Reprogramming Myeloid Cells and Restoring T Cell Fitness in Checkpoint Inhibitor Resistant Melanoma Patients

Hanna Grauers Wiktorin[1], Viktoria Ekström-Rydén[1,2], Ida Ek[1], Emma Eriksson[1,3], Tanja Lövgren[1], Clara Nordström[3], Linda C. Sandin[3], Meera R. Patel[4], Omid Hamid[5], Gustav Ullenhag[1,2,*], Angelica Loskog[1,3,*,#].

[1]Department of Immunology, Genetics and Pathology (IGP), Uppsala University; Uppsala, Sweden.

[2]Department of Oncology, Uppsala University Hospital; Uppsala, Sweden.

[3]Lokon Pharma AB, Uppsala, Sweden.

[4]Baylor College of Medicine, McNair Campus; Houston, TX.

[5]The Angeles Clinic and Research Institute, A Cedars Sinai Affiliate; Medical Oncology; Los Angeles, CA, USA.

[*]Shared senior position

[#]Corresponding author

Correspondence: angelica.loskog@igp.uu.se

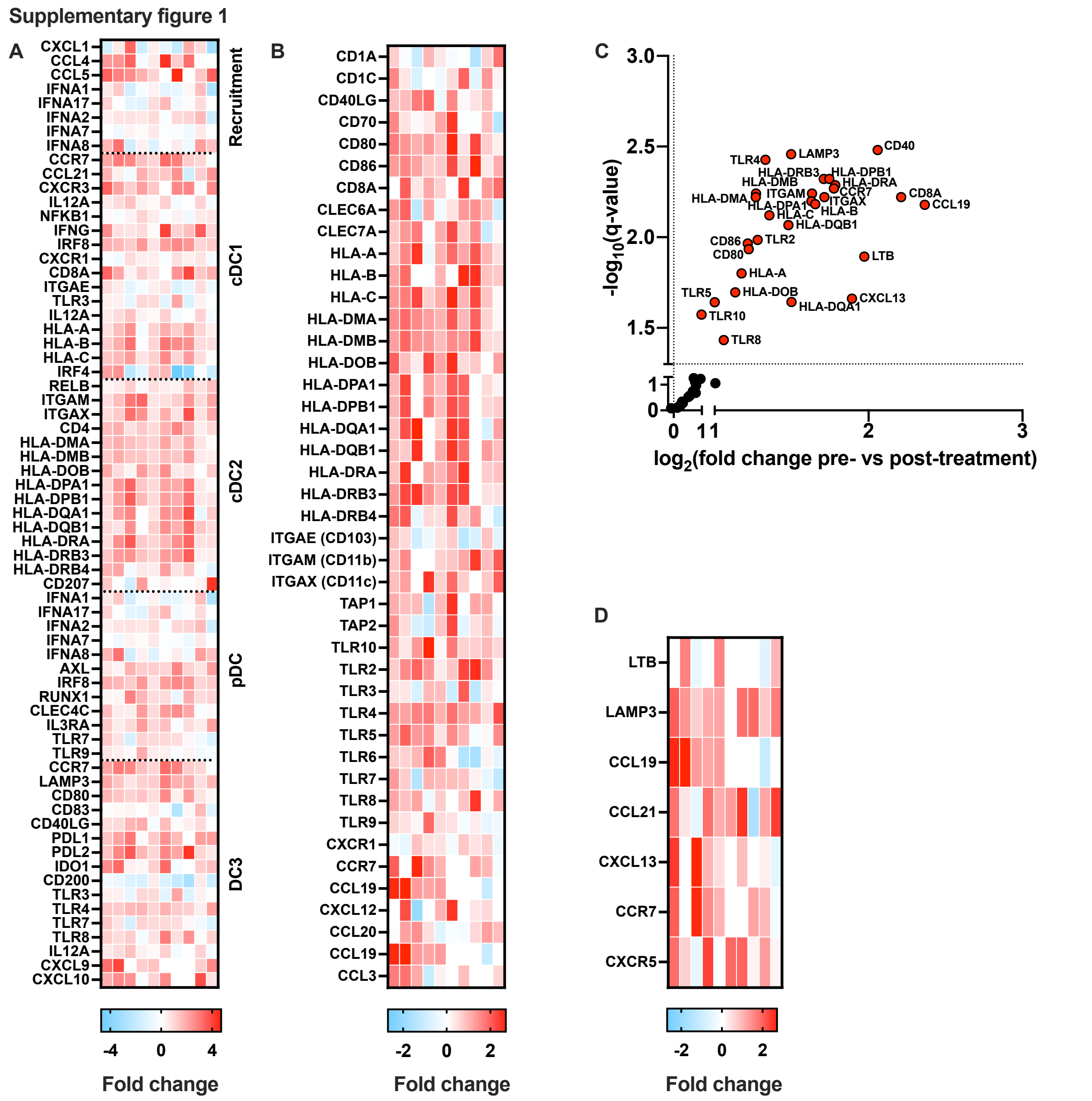

Supplementary Figure 1. LOAd703 and atezolizumab trigger elevated levels of antigen presentation, dendritic cell and tertiary lymphoid structure related genes in the tumor microenvironment. Gene expression in tumor biopsies at baseline (pre) and 27 weeks post treatment induction (post) were measured using NanoString. **(A-B**) Fold change expression of genes associated with dendritic cells (DCs: **A)** and antigen presentation (**B**). (**C**) Volcano plot of genes involved in antigen presentation, associated with DCs or teritriary lymphoid structures (TLS) in the tumor microenvironment in pre- and post-treatment (week 27 following treatment onset) samples. Red indicates genes more highly expressed post-treatment compared with pre-

treatment. Statistics by paired t tests with correction for multiple comparison by the false discovery rate (5%) method of Benjamini Hochberg. (**D**) Fold change of genes associated with TLS, post versus pre treatment. Results from gene expression are generated from 10 paired pre- and post-treatment samples, respectively.

Supplementary figure 2

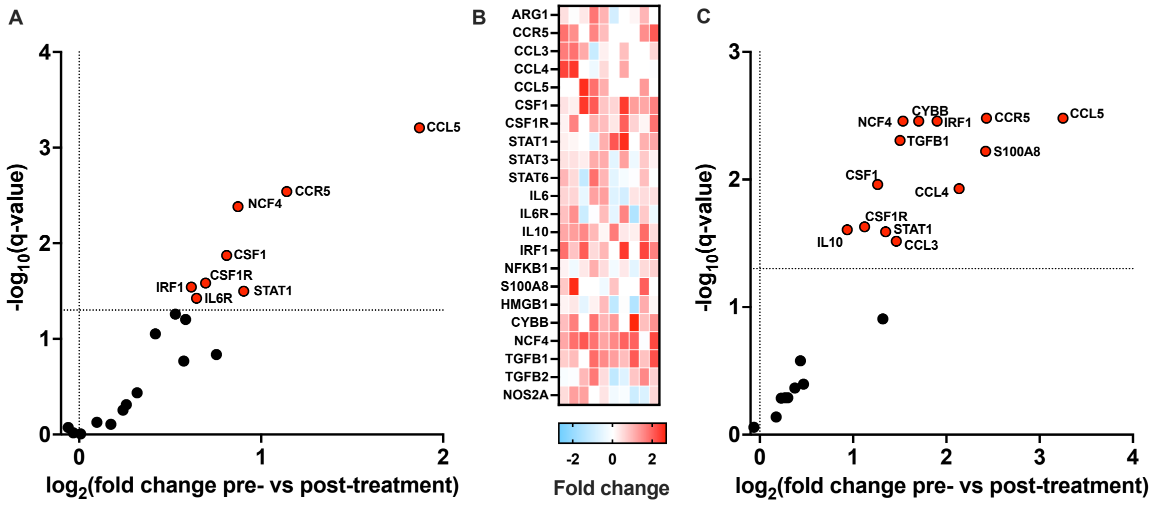

Supplementary Figure 2. LOAd703 and atezolizumab trigger elevated levels of genes associated with tumor associated macrophages and myeloid-derived suppressor cells in the tumor microenvironment. Gene expression in tumor biopsies at baseline (pre) and at nine and 27 weeks post treatment induction (post) were measured using NanoString. (**A**) Volcano plot of genes associated with tumor associated macrophages (TAMs) and myeloid-derived suppressor cells (MDSCs) in the tumor microenvironment (TME), pre-treatment versus at nine weeks post treatment induction. (**B**) Fold change gene expression of TAM/MDSC-associated genes, at baseline versus at week 27 post-treatment induction. (**C**) Volcano plot of genes associated with TAMs and MDSCs in the TME, pre-treatment versus at 27 weeks post treatment induction. In Volcano plots, red indicates genes more highly expressed post-treatment compared with pre-treatment. Statistics by paired t tests with correction for multiple comparison by the false discovery rate (5%) method of Benjamini Hochberg. Results from gene expression are generated from 15 and 10 paired pre- and post-treatment samples, week nine and 18, respectively.

Document S1: Supplementary Information

Supplementary figure 3

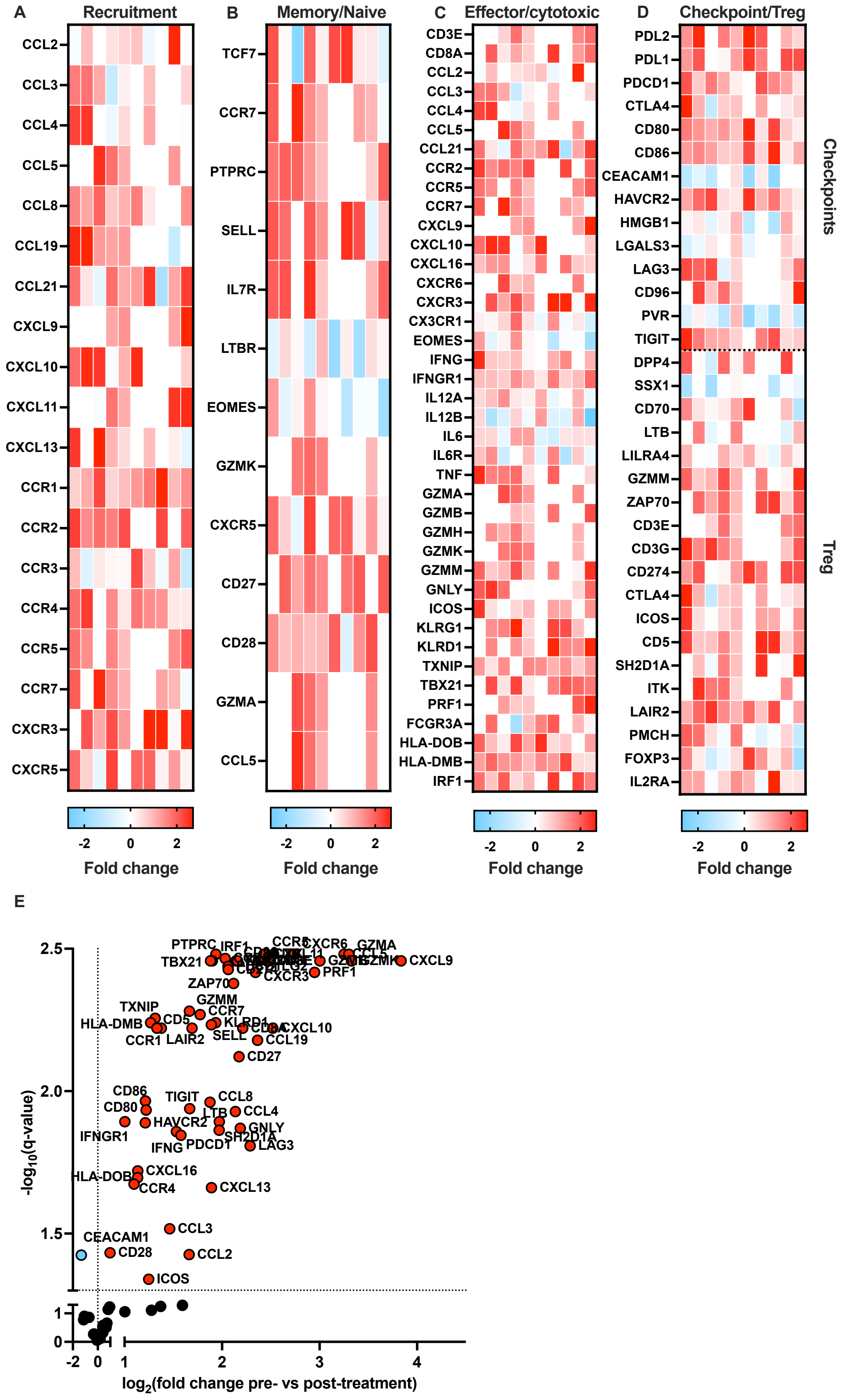

Supplementary Figure 3. LOAd703 and atezolizumab trigger elevated levels of T cell-associated genes in the tumor microenvironment. Gene expression in tumor biopsies at baseline (pre) and at 27 weeks post treatment induction (post) were measured using NanoString. (**A-D**) Fold change expression of genes associated with T cell recruitment (**A**), memory and naïve T cells (**B**), effector and cytotoxic T cells (**C**), and checkpoints (upper) and Treg (lower, **D**). (**E**) Volcano plot of T cell-associated genes in the tumor microenvironment in pre- and post-treatment samples. Red indicates genes more highly and blue genes more lowly expressed post-treatment compared with pre-treatment. Statistics by paired t tests with correction for multiple comparison by the false discovery rate (5%) method of Benjamini Hochberg. Results from gene expression are generated from 10 paired pre- and post-treatment samples, respectively.

Supplementary Figure 4

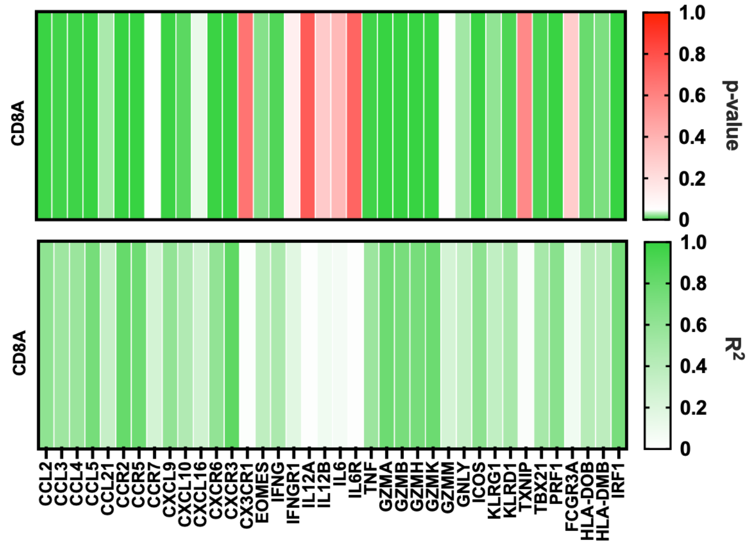

Supplementary Figure 4. Intertumoral CD8A expression strongly correlates with genes associated with T cell recruitment and effector/cytotoxic T cells. Correlations between CD8A expression and genes associated with T cell recruitment and effector/cytotoxic T cells in the tumor microenvironment measured by the NanoString technology. Statistics by linear regression, p-value upper heatmap and Pearson r (R^2) lower heatmap.

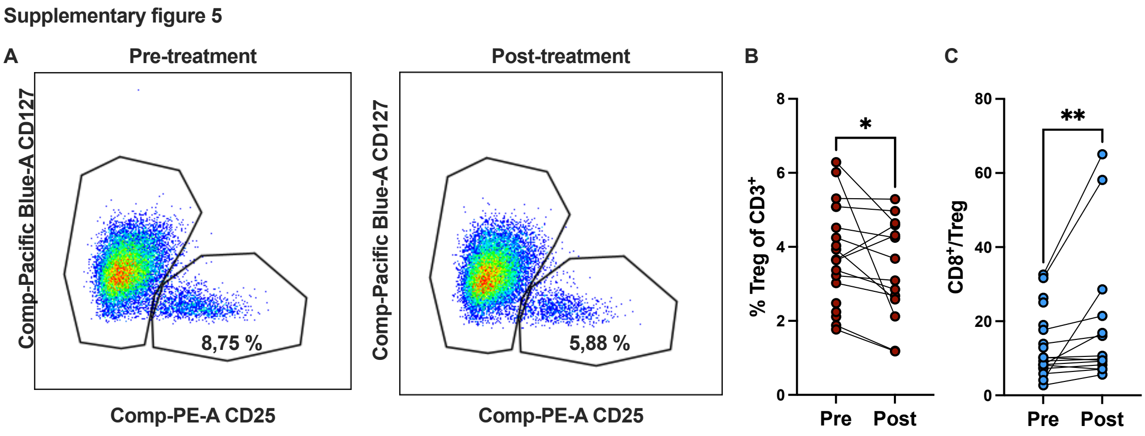

Supplementary Figure 5. Treatment with LOAd703 and atezolizumab reduce frequencies of Treg and increase the ratio between CD8^{+} T cells and Treg. (**A**) Representative dot plot of Treg in a pre- and matched post-treatment (nine weeks after treatment initiation) peripheral mononuclear blood samples measured by flowcytometry. (**B-C**) Frequencies of Treg and CD8^{+}/Treg ratio at baseline (pre) and nine weeks after (post) treatment induction. Statistics by Wilcoxon. * $p<0.05$, **$p<0.01$.

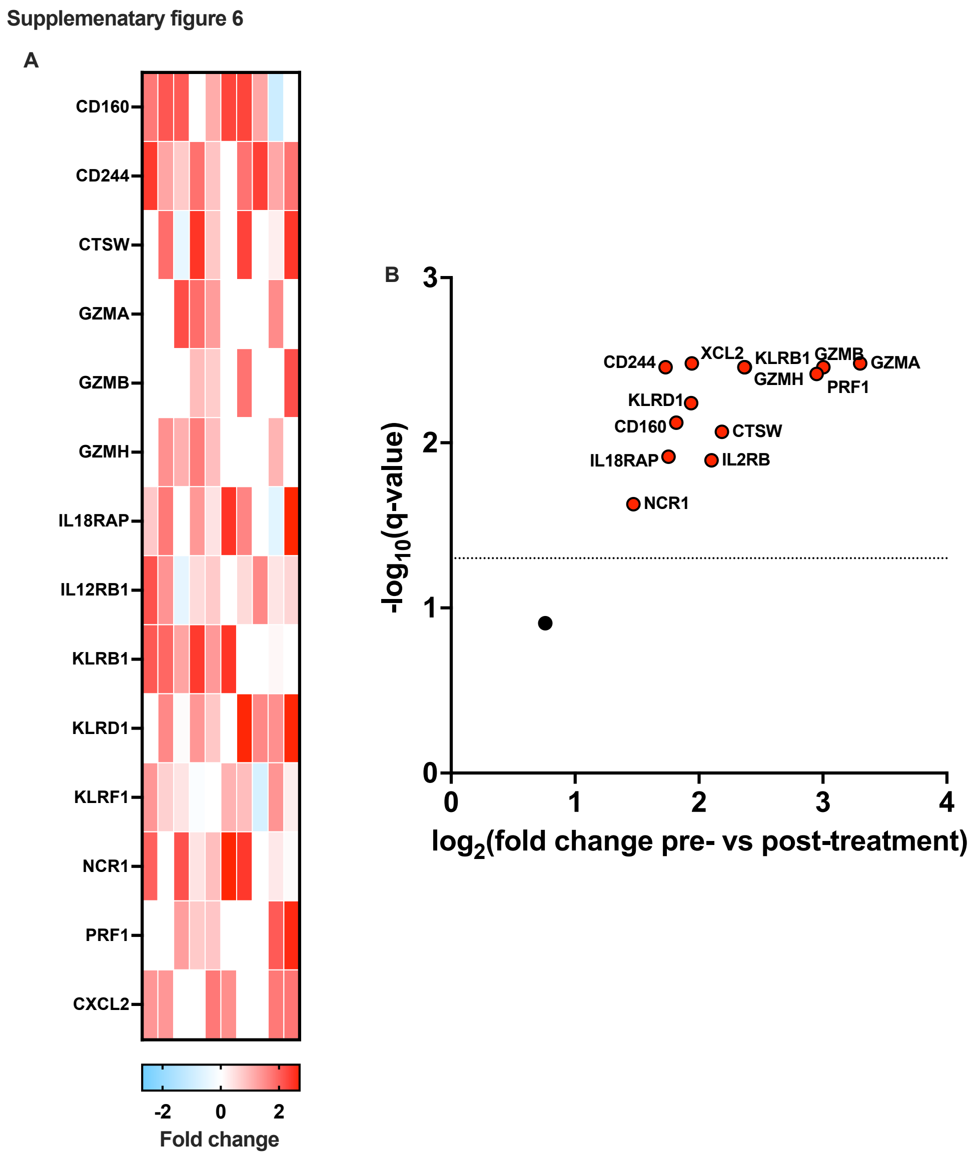

Supplementary Figure 6. LOAd703 and atezolizumab trigger elevated levels of NK cell-associated genes in the tumor microenvironment. Gene expression in tumor biopsies at baseline (pre) and at 27 weeks post treatment induction (post) were measured using NanoString. (**A**) Fold change expression of genes associated with NK cells. (**B**) Volcano plot of NK cell-associated genes in the tumor microenvironment in pre- and post-treatment samples. Red indicates genes more highly expressed post-treatment compared with pre-treatment. Statistics by paired t tests with correction for multiple comparison by the false discovery rate (5%) method of Benjamini Hochberg. Results are generated from 10 paired pre- and post-treatment samples.

Document S1: Supplementary Information

Supplementary figure 7

A

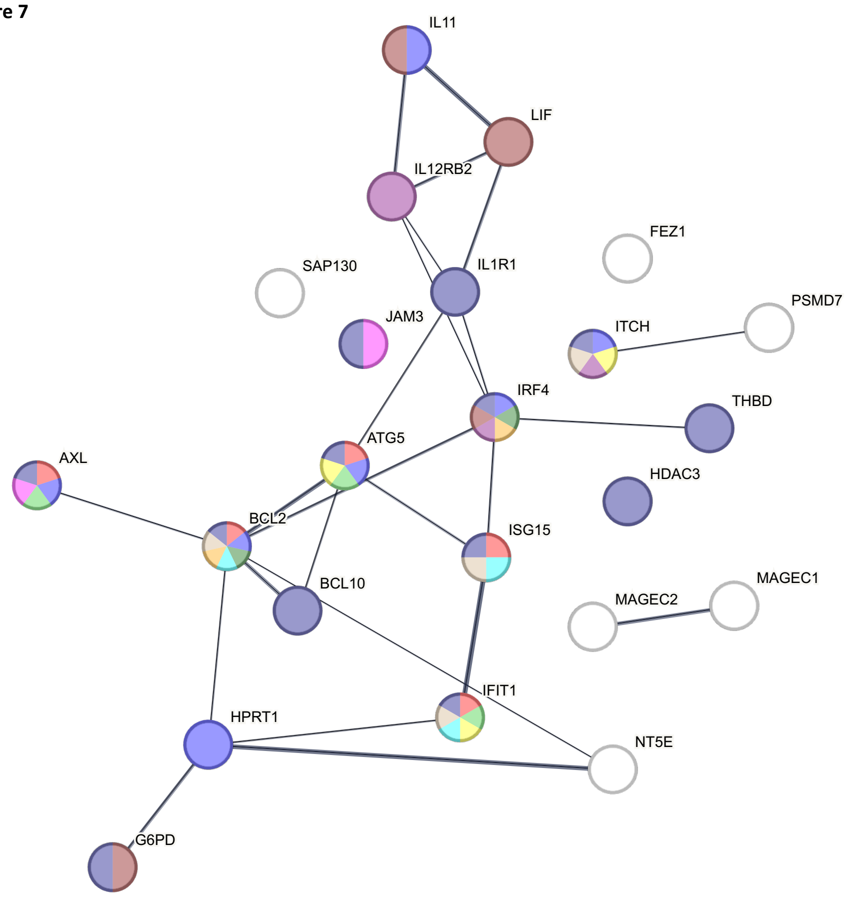

B

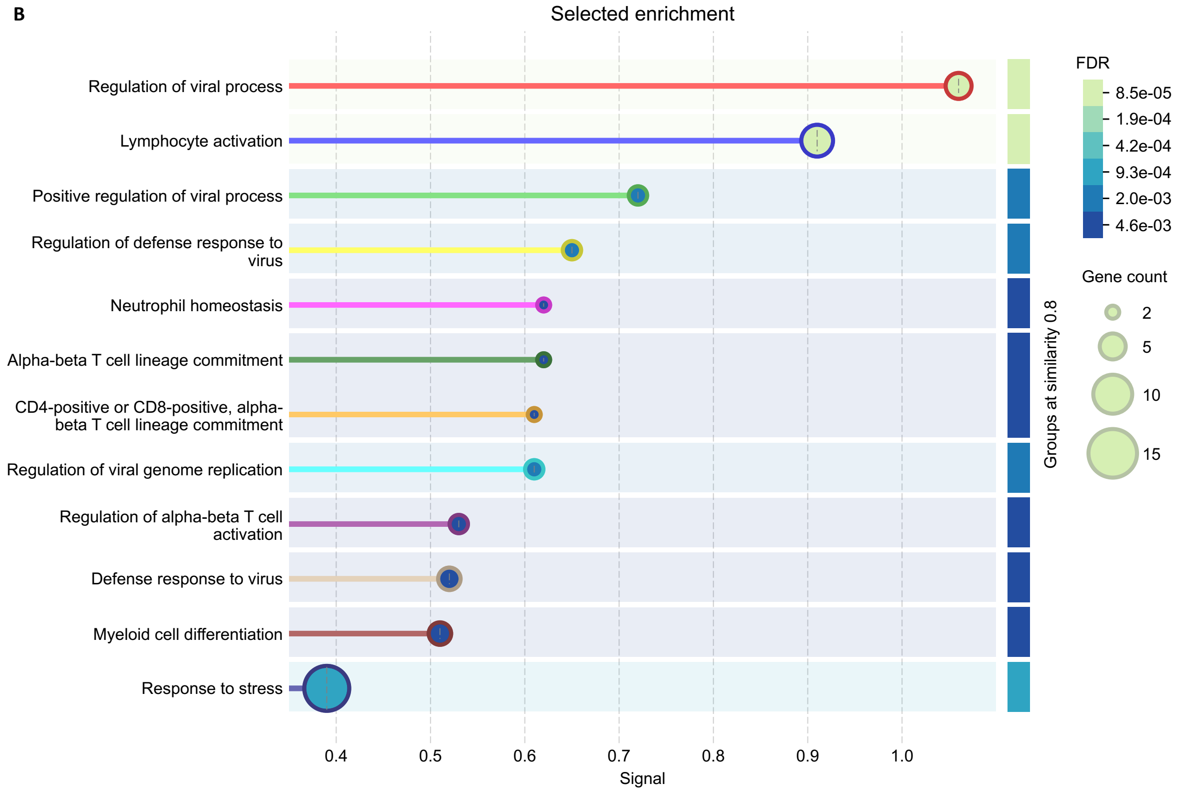

Supplementary Figure 7. Pathway analysis of pre-treatment gene expression of genes predictive of overall survival following LOAd703 and atezolizumab treatment. Gene expression in tumor biopsies at baseline were measured using NanoString platform of 770 immunomodulatory- and oncology-related genes. (**A**) Univariant Cox regression analysis were conducted for all 770 genes and proteins encoded by genes that positively or negatively impacted on overall survival were included in the gene set enrichment analysis. Proteins in **A** are color-coded based on the gene set enrichment analysis in **B**. Pathway analysis was conducted by the STRING database.

Document S1: Supplementary Information

Supplementary figure 8

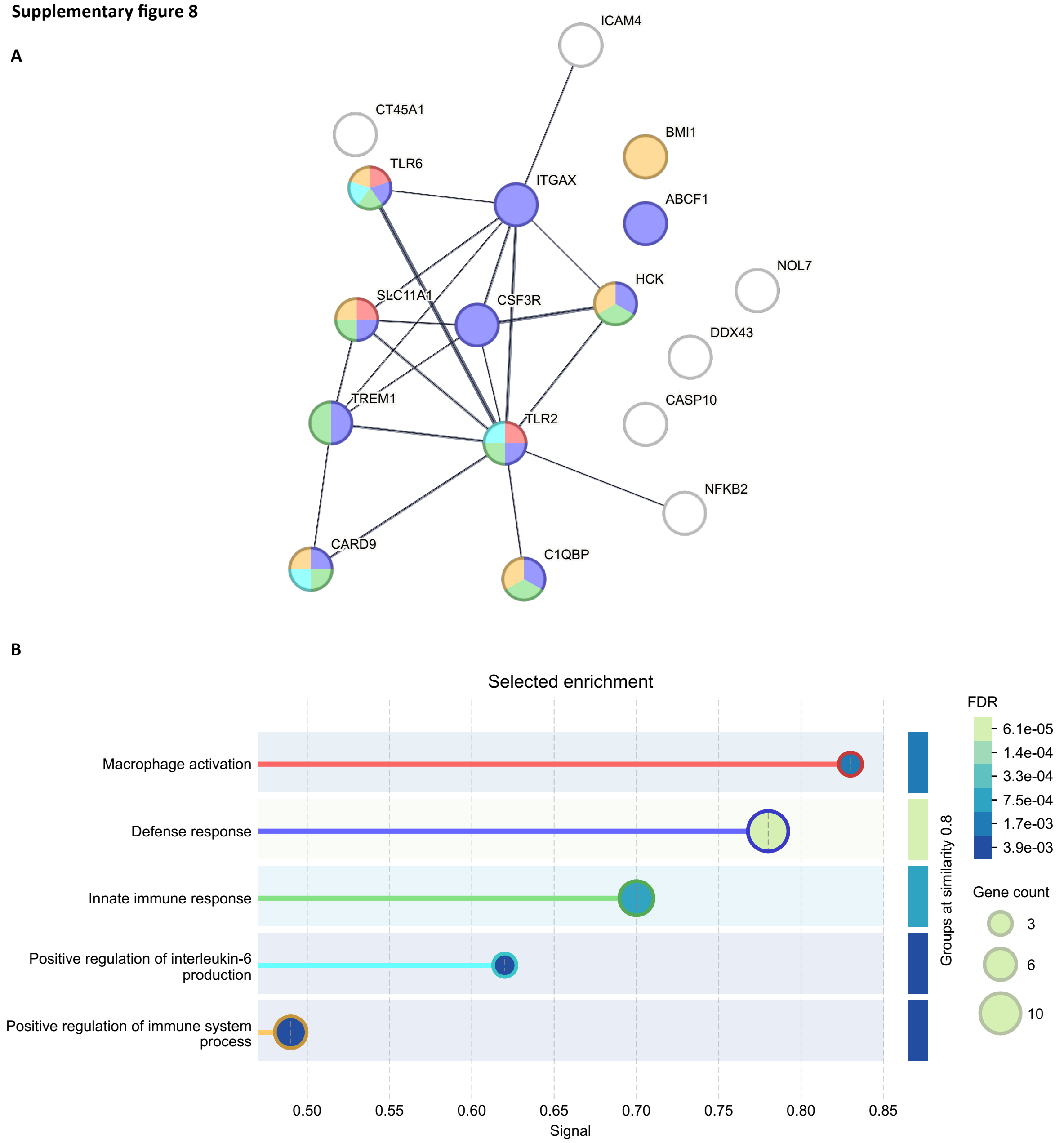

Supplementary Figure 8. Pathway analysis of post-treatment gene expression of genes predictive of overall survival following LOAd703 and atezolizumab treatment. Gene expression in tumor biopsies at nine weeks following treatment initiation were measured using NanoString platform of 770 immunomodulatory- and oncology-related genes. (**A**) Univariant Cox regression analysis were conducted for all 770 genes and proteins encoded by genes that positively or negatively impacted on overall survival were included in the gene set enrichment

analysis. Proteins in **A** are color-coded based on the gene set enrichment analysis in **B**. Pathway analysis was conducted by the STRING database.

Document S1: Supplementary Information

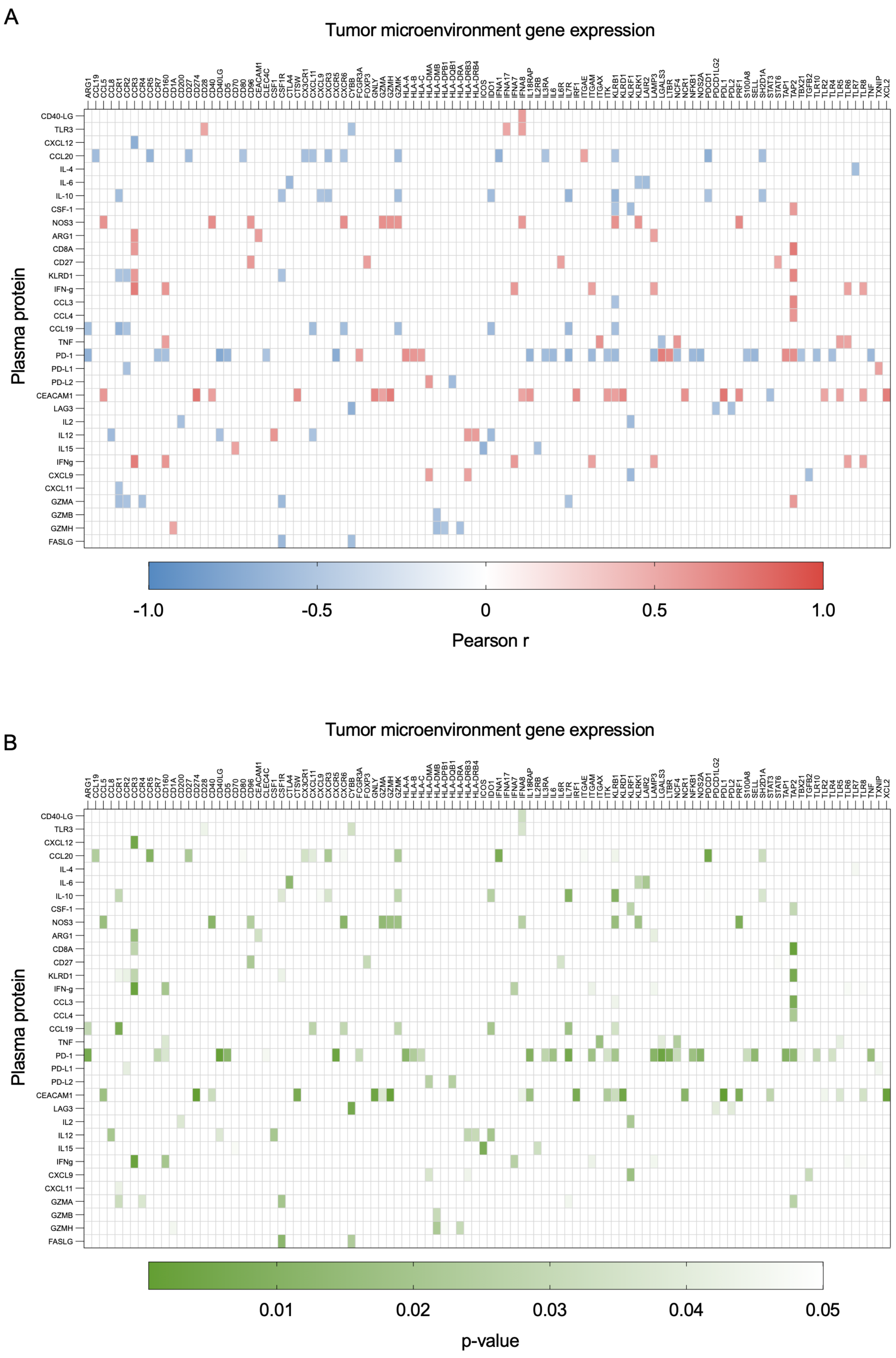

Supplementary Figure 9. Correlation between fold change (week nine versus baseline) or plasma proteins and gene expression in the tumor microenvironment. Gene expression in tumor biopsies was measured using NanoString platform of 770 immunomodulatory- and oncology-related genes and protein expression in plasma was evaluated with the Olink 96 target Immuno-oncology and Oncology II proteomics panels before and at nine weeks after treatment onset. Fold changes (FC) were calculate and FC of plasma and gene expression in the tumor microenvironment assessed by multiple linear regressions (n=15). Panels include plasma FC significantly correlated with FC of gene expression in at least one gene. (**A**) displays Pearson r, with red indicating positive and blur negative correlations, and (**B**) accompanying p-values.

Document S1: Supplementary Information

Supplementary Table 1. List of antibodies used for flow cytometric evaluation of peripheral blood mononuclear cell samples.

**Panel A**				
**Antibody**	**Fluorochrome**	**Company**	**Cat #**	**RRID**
CD16	PE	BD Biosciences	332779	AB_286828
CD56	BV421	Biolegend	318328	AB_11218798
CD3	PerCP	Biolegend	344814	AB_10639948
CD19	APC	Biolegend	363006	AB_2564128
CD14	BV510	Biolegend	301842	AB_2561946
**Panel B**				
**Antibody**	**Fluorochrome**	**Company**	**Cat #**	**RRID**
CCR6	APC	Biolegend	353416	AB_10915987
CXCR3	PE-Cy7	Biolegend	353720	AB_112119383
CD45RA	PE	Biolegend	304108	AB_314412
CCR7	BV421	BD Biosciences	562555	AB_2728119
CD3	PerCP	Biolegend	344814	AB_10639948
CD4	FITC	Biolegend	344604	AB_1937227
CD8	BV510	Biolegend	344732	AB_2564624
**Panel C**				
**Antibody**	**Fluorochrome**	**Company**	**Cat #**	**RRID**
CD25	PE	BD Biosciences	555432	AB_395826
CD27	APC	Biolegend	302810	AB_314302
ICOS	PE-Cy7	BD Biosciences	567395	AB_2916579
CD127	BV421	BD Biosciences	562436	AB_11151911
CD3	PerCP	Biolegend	344814	AB_10639948
CD4	FITC	Biolegend	344604	AB_1937227
CD8	BV510	Biolegend	344732	AB_2564624
**Panel D**				
**Antibody**	**Fluorochrome**	**Company**	**Cat #**	**RRID**
CD14	BV510	Biolegend	301842	AB_2561946
CD40	BV421	BD Biosciences	563396	AB_2738180
CD11b	APC	BD Biosciences	333143	AB_2868644
CD163	PE-Cy7	Biolegend	333614	AB_2868644
HLA-DR	PE	BD Biosciences	568230	AB_3684125
**Panel E**				
**Antibody**	**Flurochrome**	**Company**	**Cat #**	**RRID**
PD-1	APC	Biolegend	329908	AB_940475
LAG3	PE-Cy7	Biolegend	369310	AB_2629753
TIM3	BV421	Biolegend	345008	AB_11218598
4-1BB	PE	Biolegend	309804	AB_314783

CD3	PerCP	Biolegend	344814	AB_10639948
CD4	FITC	Biolegend	344604	AB_1937227
CD8	BV510	Biolegend	344732	AB_2564624

Supplementary Table 2. Significant changes in gene expression between pre- and post-(week nine) treatment tumor biopsies.

Gene	Log_2(fold change post-pre)	p-value	q-value*
GZMA	1,97	3,17E-06	2,52E-04
GZMK	2,14	3,22E-06	2,52E-04
KLRK1	1,71	1,82E-05	6,20E-04
CD96	1,71	2,26E-05	6,20E-04
HLA-DRB3	1,52	2,39E-05	6,20E-04
CCL5	1,87	1,41E-05	6,20E-04
GNLY	1,68	2,98E-05	6,64E-04
CD8A	1,54	4,17E-05	6,67E-04
PTPRC	1,26	4,08E-05	6,67E-04
HLA-DPB1	1,46	4,28E-05	6,67E-04
HLA-DPA1	1,35	9,85E-05	1,40E-03
HLA-DRA	1,39	1,22E-04	1,47E-03
SH2D1A	1,35	1,22E-04	1,47E-03
GZMH	1,31	1,34E-04	1,49E-03
CD3E	1,59	1,85E-04	1,52E-03
CD40	1,08	1,83E-04	1,52E-03
HLA-DMB	1,08	1,84E-04	1,52E-03
TXNIP	1,17	1,69E-04	1,52E-03
CYBB	1,23	1,85E-04	1,52E-03
ITK	1,33	2,38E-04	1,86E-03
HLA-DMA	0,97	2,75E-04	2,03E-03

ZAP70	1,21	2,86E-04	2,03E-03
CD27	1,44	3,63E-04	2,46E-03
CXCR3	1,5	4,20E-04	2,73E-03
CCR5	1,14	4,62E-04	2,89E-03
GZMB	1,51	4,83E-04	2,90E-03
TLR4	0,93	5,41E-04	3,11E-03
HLA-DOB	1,02	5,59E-04	3,11E-03
CTSW	1,31	5,78E-04	3,11E-03
CCL21	1,63	6,95E-04	3,51E-03
CXCL9	1,81	6,98E-04	3,51E-03
CD244	1,11	7,84E-04	3,82E-03
PRF1	1,4	8,26E-04	3,90E-03
CCR2	1,17	9,17E-04	4,14E-03
LTB	1,08	9,50E-04	4,14E-03
NCF4	0,87	9,55E-04	4,14E-03
IL2RB	1,02	1,07E-03	4,50E-03
IL7R	1,22	1,26E-03	5,17E-03
KLRD1	1,15	1,76E-03	7,03E-03
HLA-B	0,91	1,96E-03	7,66E-03
CD3G	1,19	2,44E-03	9,27E-03
CTLA4	1,11	2,63E-03	9,77E-03
HLA-C	0,76	3,20E-03	0,012
CCL19	1,4	3,29E-03	0,012
KLRG1	1,08	3,42E-03	0,012

TIGIT	1	3,94E-03	0,013
ITGAX	0,79	3,95E-03	0,013
LAIR2	0,82	4,13E-03	0,013
KLRB1	1,39	4,20E-03	0,013
CSF1	0,81	4,30E-03	0,013
IRF8	1	4,55E-03	0,014
ITGAM	0,84	5,29E-03	0,016
IFNGR1	0,73	5,35E-03	0,016
PDCD1LG2	0,98	5,41E-03	0,016
CXCL11	1,2	5,70E-03	0,016
CXCL10	1,3	6,36E-03	0,018
GZMM	0,9	6,73E-03	0,018
LAG3	1,02	6,56E-03	0,018
TBX21	0,99	6,69E-03	0,018
HLA-A	0,85	7,06E-03	0,018
ICOS	1,03	7,80E-03	0,02
CXCL13	1,35	9,24E-03	0,023
TLR2	0,72	9,45E-03	0,023
CSF1R	0,69	0,011	0,026
CXCR6	1,12	0,012	0,029
IRF1	0,62	0,012	0,029
TLR8	0,61	0,014	0,032
CD5	0,78	0,014	0,032
STAT1	0,9	0,014	0,032

CLEC4C	0,89	0,016	0,036
CEACAM1	-0,93	0,017	0,037
CD40LG	0,86	0,017	0,037
SELL	0,86	0,018	0,038
IL6R	0,65	0,018	0,038
LAMP3	0,8	0,019	0,039
XCL2	0,77	0,024	0,049
IDO1	1,05	0,025	0,05
TLR9	0,64	0,025	0,05

**post hoc test by Benjamini Hochberg with a false discovery rate of 5%.*

Supplementary Table 3. Significant changes in gene expression between pre- and post-(week 27) treatment tumor biopsies.

Gene	Log_2(fold change post-pre)	p-value	q-value*
CCL5	3,25	1,63E-04	3,31E-03
CCR5	2,43	2,07E-04	3,31E-03
CD40	2,06	1,71E-04	3,31E-03
CXCL11	2,44	2,01E-04	3,31E-03
CXCR6	2,74	2,49E-04	3,31E-03
GZMA	3,3	1,82E-04	3,31E-03
IL7R	2,43	1,21E-04	3,31E-03
IRF8	1,69	2,54E-04	3,31E-03
ITK	2,53	1,13E-04	3,31E-03
KLRK1	2,92	1,27E-04	3,31E-03
PTPRC	1,94	2,51E-04	3,31E-03
XCL2	1,94	8,18E-05	3,31E-03
CCR2	2,03	2,85E-04	3,42E-03
CD244	1,73	6,07E-04	3,49E-03
CD3E	2,48	4,83E-04	3,49E-03
CD3G	2,38	3,94E-04	3,49E-03
CD96	2,16	6,35E-04	3,49E-03
CXCL9	3,84	4,28E-04	3,49E-03
CYBB	1,7	4,92E-04	3,49E-03
GZMB	3	3,71E-04	3,49E-03
GZMH	2,37	4,89E-04	3,49E-03

GZMK	3,33	4,01E-04	3,49E-03
IRF1	1,9	5,72E-04	3,49E-03
KLRB1	2,37	6,48E-04	3,49E-03
KLRG1	2,15	4,40E-04	3,49E-03
LAMP3	1,5	6,41E-04	3,49E-03
NCF4	1,53	5,92E-04	3,49E-03
TBX21	1,88	6,25E-04	3,49E-03
TNF	2,14	5,87E-04	3,49E-03
PDCD1LG2	2,06	7,02E-04	3,65E-03
CD274	2,06	7,67E-04	3,74E-03
TLR4	1,33	7,49E-04	3,74E-03
CXCR3	2,34	8,34E-04	3,83E-03
PRF1	2,95	8,18E-04	3,83E-03
ZAP70	2,12	9,39E-04	4,19E-03
HLA-DPB1	1,75	1,13E-03	4,78E-03
HLA-DRB3	1,71	1,10E-03	4,78E-03
TGFB1	1,5	1,20E-03	4,95E-03
HLA-DRA	1,78	1,29E-03	5,17E-03
GZMM	1,67	1,34E-03	5,24E-03
CCR7	1,77	1,42E-03	5,39E-03
TXNIP	1,31	1,49E-03	5,55E-03
HLA-DMB	1,27	1,66E-03	5,75E-03
ITGAM	1,63	1,64E-03	5,75E-03
KLRD1	1,94	1,66E-03	5,75E-03

SELL	1,89	1,72E-03	5,84E-03
CCR1	1,33	1,91E-03	6,01E-03
CD5	1,69	2,04E-03	6,01E-03
CD8A	2,21	2,08E-03	6,01E-03
CXCL10	2,52	2,08E-03	6,01E-03
HLA-DMA	1,27	2,03E-03	6,01E-03
ITGAX	1,71	1,93E-03	6,01E-03
LAIR2	1,38	1,90E-03	6,01E-03
S100A8	2,42	2,02E-03	6,01E-03
HLA-DPA1	1,63	2,23E-03	6,33E-03
HLA-B	1,65	2,36E-03	6,58E-03
CCL19	2,36	2,42E-03	6,63E-03
CD160	1,82	2,81E-03	7,55E-03
CD27	2,17	2,91E-03	7,57E-03
HLA-C	1,36	2,88E-03	0,008
HLA-DQB1	1,48	3,35E-03	0,009
CTSW	2,18	3,41E-03	0,009
AXL	1,24	3,96E-03	0,01
TLR2	1,28	4,25E-03	0,01
CD86	1,21	4,51E-03	0,011
CCL8	1,88	4,68E-03	0,011
CSF1	1,26	4,70E-03	0,011
TIGIT	1,67	5,02E-03	0,012
CD80	1,22	5,15E-03	0,012

CCL4	2,14	5,29E-03	0,012
IL18RAP	1,75	5,53E-03	0,012
IL2RB	2,1	5,89E-03	0,013
IFNGR1	1	6,01E-03	0,013
LTB	1,97	6,07E-03	0,013
HAVCR2	1,21	6,21E-03	0,013
GNLY	2,19	6,58E-03	0,013
SH2D1A	1,97	6,77E-03	0,014
PDCD1	1,53	6,93E-03	0,014
IFNG	1,58	7,24E-03	0,014
LAG3	2,29	7,98E-03	0,016
HLA-A	1,17	8,23E-03	0,016
CXCL16	1,14	0,01	0,019
HLA-DOB	1,13	0,011	0,02
CLEC4C	1,23	0,011	0,021
IDO1	1,8	0,012	0,021
CCR4	1,1	0,012	0,021
CXCL13	1,89	0,012	0,022
IL3RA	1,04	0,013	0,023
HLA-DQA1	1,5	0,013	0,023
TLR5	1	0,013	0,023
CSF1R	1,12	0,014	0,023
NCR1	1,47	0,014	0,024
IL10	0,94	0,015	0,025

STAT1	1,35	0,015	0,026
TLR10	0,99	0,016	0,027
CCL3	1,46	0,019	0,03
CD28	0,98	0,023	0,037
TLR8	1,06	0,023	0,037
CCL2	1,66	0,024	0,037
CD200	-1,12	0,024	0,037
CEACAM1	-1,32	0,024	0,038
ICOS	1,25	0,03	0,046

post hoc test by Benjamini Hochberg with a false discovery rate of 5%.

Supplementary Table 4. Significant alterations in protein levels of T cell related proteins and checkpoints between pre- and post- (week nine) treatment serum samples.

Protein	Log_2(fold change post-pre)	p-value	q-value*
PD-L1	4,13	1,84E-13	4,24E-12
CXCL9	0,84	0,004	0,034
CD27	0,31	0,004	0,034
TNF	0,31	0,009	0,038
CXCL11	0,50	0,010	0,038
CXCL13	0,45	0,011	0,038
CXCL10	0,66	0,013	0,038
CD8A	0,57	0,014	0,038
PD-1	0,55	0,015	0,038
GZMA	0,49	0,017	0,040
KLRD1	0,47	0,022	0,045

**post hoc test by Benjamini Hochberg with a false discovery rate of 5%.*

Supplementary Table 5. Significant alterations in protein levels of T cell related proteins between pre- and post- (week 18) treatment serum samples.

Protein	n	p-value*	q-value#
CD8A	12	0.049	0.13
CD27	12	0.023	0.11
IL-12	12	0.032	0.11
GZMH	13	0.029	0.11
CXCL9	12	0.017	0.11
CXCL10	12	0.035	0.11
TNF	12	0.019	0.11

**Paried t test.*

# post hoc test by Benjamini Hochberg with a false discovery rate of 5%.

Supplementary Table 6. Significant alterations in serum protein levels of biomarkers for NK cells between pre- and post- (nine weeks) treatment samples.

Protein	Log_2(fold change post-pre)	p-value	q-value*
CXCL9	0,84	3,51E-03	0,029
IL18	0,31	4,88E-03	0,029
CXCL11	0,50	0,010	0,039
CXCL10	0,66	0,013	0,039
GZMA	0,49	0,017	0,042

**post hoc test by Benjamini Hochberg with a false discovery rate of 5%.*
